# Diethyl Phthalate (DEP) as a potential osteosarcoma risk factor: a multi-omics study integrating network Toxicology, single-cell RNA sequencing, and molecular docking

**DOI:** 10.1080/14756366.2025.2611582

**Published:** 2026-02-16

**Authors:** Shangqi Yin, Wuzheng Liu, Chunxiao Gao, Chunyan Li, Jun Wu

**Affiliations:** Department of Clinical Laboratory, Beijing Jishuitan Hospital, Capital Medical University, Beijing, PR China

**Keywords:** Diethyl phthalate, osteosarcoma, network toxicology, molecular docking, P4HA2

## Abstract

Diethyl phthalate (DEP), a common plasticiser and endocrine disruptor, has been linked to cancer, but its role in osteosarcoma (OS) remains unclear. This study integrated network toxicology, transcriptomics, protein-protein interaction (PPI) analysis, machine learning, molecular docking, molecular dynamics (MD), single-cell RNA sequencing (scRNA-seq), and external validation to investigate DEP-related mechanisms in OS. We identified 45 DEP-responsive genes enriched in extracellular matrix-related pathways. PPI network analysis revealed 11 hub genes, of which LASSO, SVM-RFE, and Boruta algorithms consistently prioritised P4HA2, COL18A1, and COL10A1. Docking and MD simulations supported stable binding of DEP to P4HA2 and COL18A1 via hydrogen bonds and hydrophobic interactions. scRNA-seq demonstrated celltype-specific expression of these genes. Validation cohorts confirmed their upregulation in OS, with AUC values up to 0.950. These findings suggest that DEP may promote OS progression by targeting extracellular matrix remodelling, offering new diagnostic biomarkers and hypothesis-generating evidence for environmental osteocarcinogenesis.

## Inflammation

In recent years, the widespread use of chemical materials in daily life has raised increasing concerns about their potential threats to human health. Among them, plasticisers are extensively used as chemical additives to enhance the flexibility and processability of plastic products. Diethyl phthalate (DEP), one of the most commonly used plasticisers, is found in a wide range of everyday items including toys, toothbrushes, tool handles, automobile parts, medical devices, food packaging, and bottle caps^1^. Additionally, DEP is commonly incorporated into personal care products such as cosmetics, perfumes, hair sprays, soaps, and cleaning agents. This ubiquity results in inevitable human exposure to DEP^2^. Despite regulatory restrictions on certain phthalates in the European Union, DEP continues to circulate widely as a so-called “low-toxicity” alternative. A study estimated that approximately 13 million children under the age of six in the United States may be exposed to potentially hazardous chemicals, including DEP, during critical developmental windows^3^. Owing to its high lipophilicity and environmental persistence, DEP tends to bioaccumulate in organisms, raising concerns about its long-term reproductive and carcinogenic risks, particularly in children. While DEP is often regarded as less toxic than other phthalates, its potential health effects following chronic exposure have gained increasing scientific attention^4^. However, its role in tumorigenesis remains largely unexplored, warranting further investigation.

Osteosarcoma (OS) is a highly aggressive bone malignancy originating from mesenchymal stem cells, predominantly affecting children and adolescents^5^. It is recognised as the most common primary malignant bone tumour in the paediatric population. Due to its insidious onset, OS is often diagnosed at an advanced stage, by which time significant local invasion and distant metastases-particularly to the lungs-may have already occurred, severely compromising patient outcomes^6^. Although multimodal treatment combining surgery and chemotherapy has improved prognosis to some extent, the 5-year overall survival rate has remained stagnant over the past decades.

OS development involves a complex interplay of genetic susceptibility, radiotherapy, and environmental factors^7^. In recent years, increasing attention has been paid to the potential role of environmental pollutants in tumorigenesis. Bisphenol A (BPA), a well-known endocrine-disrupting compound, has been shown to promote OS cell proliferation, migration, and invasion by regulating DLGAP5 expression and activating the IL-6/JAK2/STAT3 signalling pathway^8^. Upregulation of BPA-related genes has also been linked to the formation of an immunosuppressive tumour microenvironment, potentially exacerbating disease progression. Phthalates, such as DEP, are widely used plasticisers known to interfere with endocrine signalling. Large-scale epidemiological data suggest that phthalate exposure in childhood may be associated with increased incidence of OS and lymphoma before age 19, although the molecular mechanisms remain unclear^9^. A structurally similar compound, butyl benzyl phthalate (BBP), has been reported to inhibit ATP-induced proliferation of human osteosarcoma HOS cells, highlighting potential toxicity risks during bone remodelling^10^. Moreover, DEP and its metabolite monoethyl phthalate (MEP) have been associated with cancer-specific mortality in breast cancer patients^11^. However, no study has systematically investigated the molecular mechanisms by which DEP may contribute to OS pathogenesis.

To address the current knowledge gap, we integrated network toxicology, machine learning, molecular docking, molecular dynamics (MD), and single-cell RNA sequencing (scRNA-seq) to investigate the potential oncogenic mechanisms of DEP in OS. We comprehensively profiled DEP-related targets by combining multiple chemical-gene interaction databases, including CTD, ChEMBL, SwissTargetPrediction, and TargetNet, with OS transcriptomic datasets. Core candidates were identified via intersection with DEGs and further refined using three complementary machine learning algorithms: LASSO, SVM-RFE, and Boruta. Molecular docking analysis and MD simulations were employed to assess the binding potential of DEP to hub proteins. Additionally, scRNA-seq data were analysed to explore spatial distribution patterns of candidate genes within the OS tumour microenvironment. This integrative multi-omics strategy provides a holistic mechanistic framework linking environmental pollutants to cancer biology, offering novel insights into environmental aetiology and therapeutic biomarker discovery in OS.

## Methods and materials

### Identification and collection of DEP associated targets

The canonical structure, molecular formula, and SMILES notation [CCOC(=O)c1ccccc1C(=O)OCC] of DEP were obtained from the PubChem database (https://pubchem.ncbi.nlm.nih.gov/)^12^ using the compound name “Diethyl Phthalate”. DEP associated targets were then retrieved from four publicly available resources, included the Comparative Toxicogenomics Database (CTD) (https://ctdbase.org/)^13^, ChEMBL (https://www.ebi.ac.uk/chembl/)^14^, TargetNet (http://targetnet.scbdd.com)^15^, and SwissTargetPrediction (http://www.swisstargetprediction.ch/)^16^ database. Initially, we queried the CTD and ChEMBL databases using the keyword “Diethyl Phthalate” and restricted the search scope to “Homo sapiens”, thereby retrieving experimentally validated or annotated human gene targets. Subsequently, the SMILES structure of DEP was submitted to the TargetNet platform, with the inclusion models set to “AUC ≥ 0.7” and “ECFP4 fingerprints” selected as the Fingerprint type. This allowed for the prediction of high-confidence targets based on molecular similarity and model performance. Additionally, the same SMILES structure was uploaded to the SwissTargetPrediction database with species restricted to Homo sapiens to identify further potential targets. All collected gene targets from the four databases were integrated and deduplicated. Gene names were standardised using the UniProt database (https://www.uniprot.org/)^17^, and a comprehensive list of DEP associated genes was compiled as the reference gene set for subsequent network construction and functional enrichment analyses.

### Data acquisition and processing

Three independent OS gene expression datasets, GSE99671, GSE39058 and GSE19276, were obtained from the Gene Expression Omnibus (GEO) database (https://www.ncbi.nlm.nih.gov/). GSE99671 is a high-throughput RNA-sequencing dataset based on the GPL20148 platform, containing 18 OS tissue samples and 18 normal bone tissue controls. GSE39058 is a microarray dataset based on the GPL14951 platform, comprising 37 OS samples and 10 adjacent normal tissue samples. Raw data from GSE99671 were preprocessed using the DESeq2 package, and GSE39058 using the limma package^18^, for background correction, normalisation, and log2 transformation. Batch effects between the datasets were adjusted using the sva package^19^, and the two datasets were merged to generate a unified training cohort for downstream analyses. The GSE19276 dataset, based on the GPL6848 microarray platform, comprises 23 OS samples and 5 non-malignant bone tissue samples. It was employed as an independent validation set to evaluate the expression levels and potential functional roles of the identified hub genes. To further examine the expression distribution and functional roles of hub genes at single-cell resolution, we analysed scRNA-seq data from the GSE162454 dataset, which includes six OS samples. The data were processed using the Seurat (v5.2.1) package^20^, including quality control, dimensionality reduction, clustering, and cell-type annotation. The resulting single-cell atlas was used to visualise gene expression across different cell populations and to explore potential biological functions in the OS microenvironment.

### Identification and functional enrichment analysis of differentially expressed genes (DEGs) in OS

The merged expression matrix from the training cohort was used to identify DEGs between OS tissues and control tissues. Principal component analysis (PCA) was first performed using the ggplot2 package^21^ to assess the distribution of samples and batch effects. Differential gene expression analysis was then conducted using the limma package in R, with the threshold set as |log2 fold change| > 0.5 and *p* values < 0.05. To visualise the expression patterns and distribution of DEGs, volcano plots were generated using ggplot2, and heatmaps of the top 50 DEGs were constructed using the pheatmap^22^ package. Functional annotation and enrichment analysis of the DEGs were carried out using the clusterProfiler and GOplot packages. Gene Ontology (GO) analysis included enrichment in three categories: biological processes (BP), cellular components (CC), and molecular functions (MF). Additionally, gene set enrichment analysis (GSEA) was performed with the clusterProfiler^23^ and enrichplot^24^ packages, using the org.Hs.eg.db annotation package as the reference gene set. This allowed for the assessment of pathway-level trends across the entire transcriptome.

### Protein-protein interaction (PPI) network construction and hub gene identification

Key candidate genes implicated in the toxicological mechanisms of DEP in OS were identified by intersecting DEGs with predicted DEP-related targets using Venn diagram analysis. The intersection was visualised using the circlize^25^ and ComplexHeatmap^26^ packages in R. GO and KEGG enrichment analyses were conducted for the 45 overlapping genes using the clusterProfiler package in R. KEGG enrichment was performed via the enrichKEGG function with parameters set for Homo sapiens (organism = “hsa”), and adjusted *p* values (*p*.adjust < 0.05) were considered statistically significant. The overlapping genes were then submitted to the STRING database (https://string-db.org/) to retrieve protein–protein interaction (PPI) information, with the organism set to “Homo sapiens” and the minimum required interaction score set at 0.4 (medium confidence). The resulting network was exported and further analysed using Cytoscape^27^ software (version 3.10.3). Isolated nodes without connections were removed, resulting in a refined PPI network containing 33 connected nodes. To identify the most critical hub genes within the network, we employed the cytoHubba^28^ plugin in Cytoscape and applied six commonly used topological analysis algorithms: Maximal Clique Centrality (MCC), Maximum Neighbourhood Component (MNC), Degree, Density of Maximum Neighbourhood Component (DMNC), Edge Percolated Component (EPC), and BottleNeck^28^. Genes ranking highly across multiple algorithms were selected as candidate hub genes for subsequent validation and functional analysis.

### Feature gene selection using machine learning algorithms

To refine the key gene candidates identified from the PPI network analysis, we employed three machine learning algorithms to perform robust feature selection: Least Absolute Shrinkage and Selection Operator (LASSO), Support Vector Machine-Recursive Feature Elimination (SVM-RFE), and the Boruta feature selection algorithm. LASSO regression was implemented using the glmnet^29^ package in R, with a logistic regression model and 10-fold cross-validation to determine the optimal regularisation parameter (λ). Genes with non-zero coefficients at the optimal λ value were selected as important features. SVM-RFE was performed using the caret^30^ and e1071^31^ packages. A repeated 5-fold cross-validation strategy (repeats = 3) was adopted to evaluate classification accuracy, and recursive elimination was applied based on feature importance scores to select top-ranked genes. The Boruta algorithm was run using the Boruta package^32^ in R. Based on a random forest classifier, Boruta iteratively compared the importance of each real feature to its permuted shadow counterpart and retained only those classified as “Confirmed” with consistently high relevance. The final set of core feature genes was obtained by intersecting the results of the three machine learning methods and was used for subsequent expression validation and functional analysis.

### ScRNA-seq analysis of core genes in OS samples

The GSE162454 scRNA-seq dataset, comprising six OS samples, was analysed to profile the cellular distribution of DEP-associated hub genes in the tumour microenvironment. Raw data processing and downstream analyses were conducted in R (version 4.4.1) using the Seurat package (version 5.2.1) ^33^. Quality control was performed by filtering out cells with < 200 or > 7,500 detected genes, mitochondrial gene percentage > 10%, or total RNA counts >1,000, thereby removing low-quality and dying cells. The filtered matrix was normalised using the LogNormalize method, and the top 2,000 highly variable genes (HVGs) were identified with the FindVariableFeatures function. PCA was applied for dimensionality reduction, and Harmony integration was used to correct batch effects across samples. The first 15 principal components were used to construct a shared nearest neighbour (SNN) graph, followed by unsupervised clustering via the FindClusters function at a resolution of 0.5. Two-dimensional visualisation was achieved using Uniform Manifold Approximation and Projection (UMAP). Cell type annotation was carried out through a combination of automated labelling using the SingleR package and manual verification based on canonical lineage markers. Clusters were assigned to major immune and stromal cell populations, including macrophages, CD8^+^ T cells, fibroblasts, endothelial cells, B cells, monocytes, astrocytes, and haematopoietic stem cells (HSCs). Expression distributions of the three hub genes (COL18A1, P4HA2, and COL10A1) across cell types were visualised using FeaturePlot, VlnPlot, and DotPlot functions. These visualisations provided insights into their cellular localisation and functional relevance within the OS microenvironment.

### Molecular docking and molecular simulations analysis for DEP and core targets

Molecular docking and molecular simulations were conducted to evaluate the binding affinities and interaction modes between DEP and the three selected hub proteins. Protein structure information for each core gene was retrieved from the UniProt database using their respective Entry IDs. Corresponding crystal structures were then downloaded from the RCSB Protein Data Bank (https://www.rcsb.org/)^34^ in PDB format. The PDB IDs for COL18A1, COL10A1, and P4HA2 are 3HSH, 1GR3, and 7ZSC, respectively. The 3D structure of DEP was obtained from the PubChem database in SDF format and converted to PDB format using Open Babel (version 3.1.1) software^35^. The geometry of the DEP molecule was further optimised using Chem3D (version 23.1.1.3) to identify its lowest energy conformation. Receptor protein preprocessing was conducted using PyMOL (version 3.0.3) by removing water molecules and ions to yield clean protein structures. Preparation for docking was performed using AutoDockTools (version 1.5.7), which included the addition of polar hydrogen atoms, assignment of Gasteiger charges, and identification of torsion bonds. Both protein and ligand structures were saved in pdbqt format. Binding pockets were either defined based on Protenix (https://www.biorxiv.org/content/10.1101/2025.01.08.631967v1) prediction with appropriate grid box size and centre coordinates. Docking simulations were conducted using AutoDock Vina (version 1.1.2), and the binding affinity (kcal/mol) for each protein-ligand complex was recorded. The conformation with the lowest binding energy was selected for further analysis.

All-atom molecular dynamics (MD) simulations were carried out using GROMACS 2023, employing the Amber ff14SB force field for the protein and GAFF2 for the ligand, in conjunction with the TIP3P water model. Each system was first subjected to energy minimisation, followed by equilibration under NVT and NPT ensembles. Production runs were then performed for 100 ns at 300 K, utilising a Langevin thermostat for temperature control and a Berendsen barostat for pressure regulation. Binding free energies were estimated via the MM-PBSA method using Gmx_MMPBSA v1.5.5 (https://pubs.acs.org/doi/10.1021/acs.jctc.1c00645), based on the final 50 ns of each trajectory. Principal component analysis (PCA) and free energy landscape (FEL) calculations were conducted on the production trajectories to characterise dominant low-energy conformational states. Protein-ligand interactions were analysed using ProLIF (https://doi.org/10.1186/s13321-021-00548-6). All trajectory analyses were performed with built-in GROMACS utilities, and molecular visualisations were generated using PyMOL. Binding affinities and molecular interactions were used to assess the potential of DEP as a functional ligand for each target protein, thereby providing mechanistic insights into its toxicological effects in OS.

### Validation of hub gene expression using independent dataset

To validate the expression of hub genes, we utilised the GSE19276 dataset, which consists of 23 OS and 5 non-malignant bone tissue samples based on the GPL6848 microarray platform. Normalisation and log2 transformation were applied. Differential expression and ROC curve analyses were conducted to assess diagnostic performance of hub genes.

## Results

### Identification and visualisation of DEGs in OS

**Figure 1.**
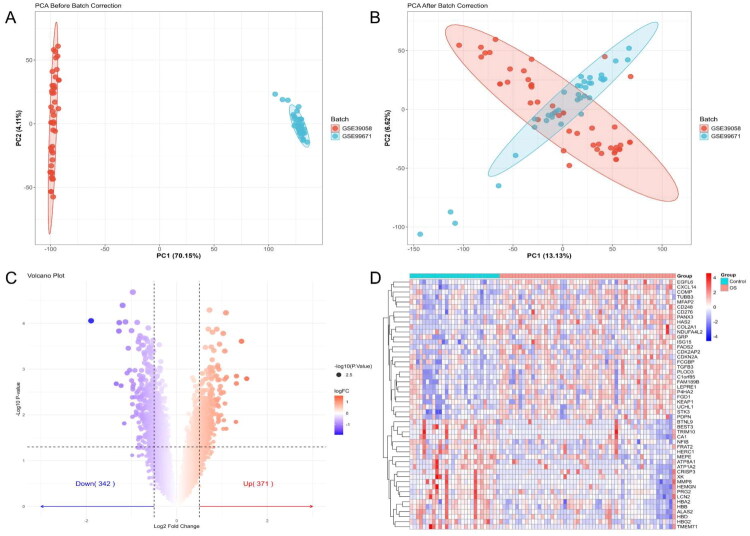
Identification and visualisation of DEGs in OS samples. (A) PCA before batch effect correction. (B) PCA after batch effect correction using the sva package. (C) Volcano plot of DEGs (|Log2 fold change| > 0.5, *p*-value < 0.05). (D) Heatmap displaying the top 50 upregulated and downregulated genes between OS and control tissues.

Batch effects between datasets were minimised by integrating and normalising two GEO gene expression profiles, GSE99671 and GSE39058. PCA revealed that the pre-correction samples clustered into two distinct groups, indicating a strong batch effect. In contrast, the post-correction samples showed a well-intermixed distribution, suggesting that the batch bias was effectively removed, and the data were suitable for downstream analysis (Figure 1A, 1B). Differential expression analysis was then performed on the corrected dataset using the limma package, with thresholds set at |log2 fold change| > 0.5 and *P* value < 0.05. A total of 713 DEGs were identified provided in Supplementary Table S1, including 371 upregulated and 342 downregulated genes (Figure 1C). Heatmap visualisation of the top 50 most variably expressed DEGs revealed distinct clustering patterns between OS and normal tissue samples, reflecting substantial transcriptional differences (Figure 1D).

### Functional enrichment analysis of DEGs

**Figure 2.**
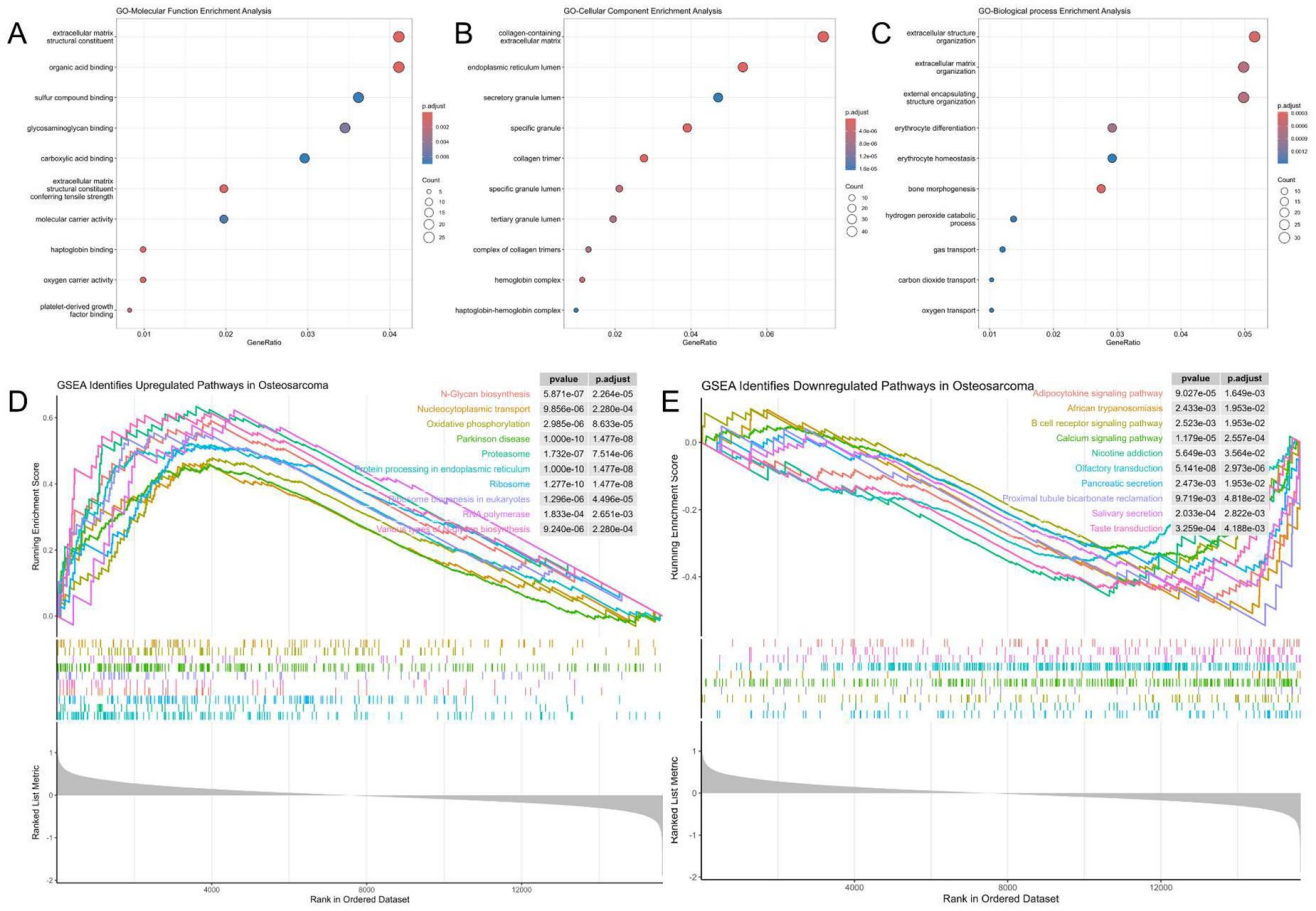
Functional enrichment analysis of DEGs. (A–C) GO enrichment results for Molecular Function (MF), Cellular Component (CC), and Biological Process (BP). (D) GSEA enrichment of upregulated DEGs. (E) GSEA enrichment of downregulated DEGs. Enrichment analysis was performed using the clusterProfiler package; terms with adjusted *p*-value < 0.05 were considered significant.

GO enrichment analysis revealed that in the MF category, DEGs were primarily enriched in extracellular matrix (ECM) structural constituent, organic acid binding, sulphur compound binding, glycosaminoglycan binding, and molecular carrier activity (Figure 2A). CC terms were mainly associated with the collagen-containing ECM, endoplasmic reticulum lumen, secretory granules, haemoglobin complex, and haptoglobin–haemoglobin complex (Figure 2B). BP terms included ECM organisation, erythrocyte differentiation and homeostasis, bone morphogenesis, and oxygen transport, indicating potential roles in matrix remodelling and haematologic functions (Figure 2C). GSEA based on the entire transcriptome further highlighted pathway-level trends. Downregulated pathways included adiponectin signalling, B cell receptor signalling, calcium signalling, and several secretion-related pathways, suggesting suppressed immune and metabolic activity in OS tissues (Figure 2D). In contrast, upregulated genes were significantly enriched in N-glycan biosynthesis, nucleocytoplasmic transport, oxidative phosphorylation, proteasome activity, ribosome biogenesis, and endoplasmic reticulum protein processing, reflecting enhanced protein synthesis and metabolic adaptation in tumour cells (Figure 2E).

### Integration of DEGs and DEP-related targets and functional enrichment analysis

**Figure 3.**
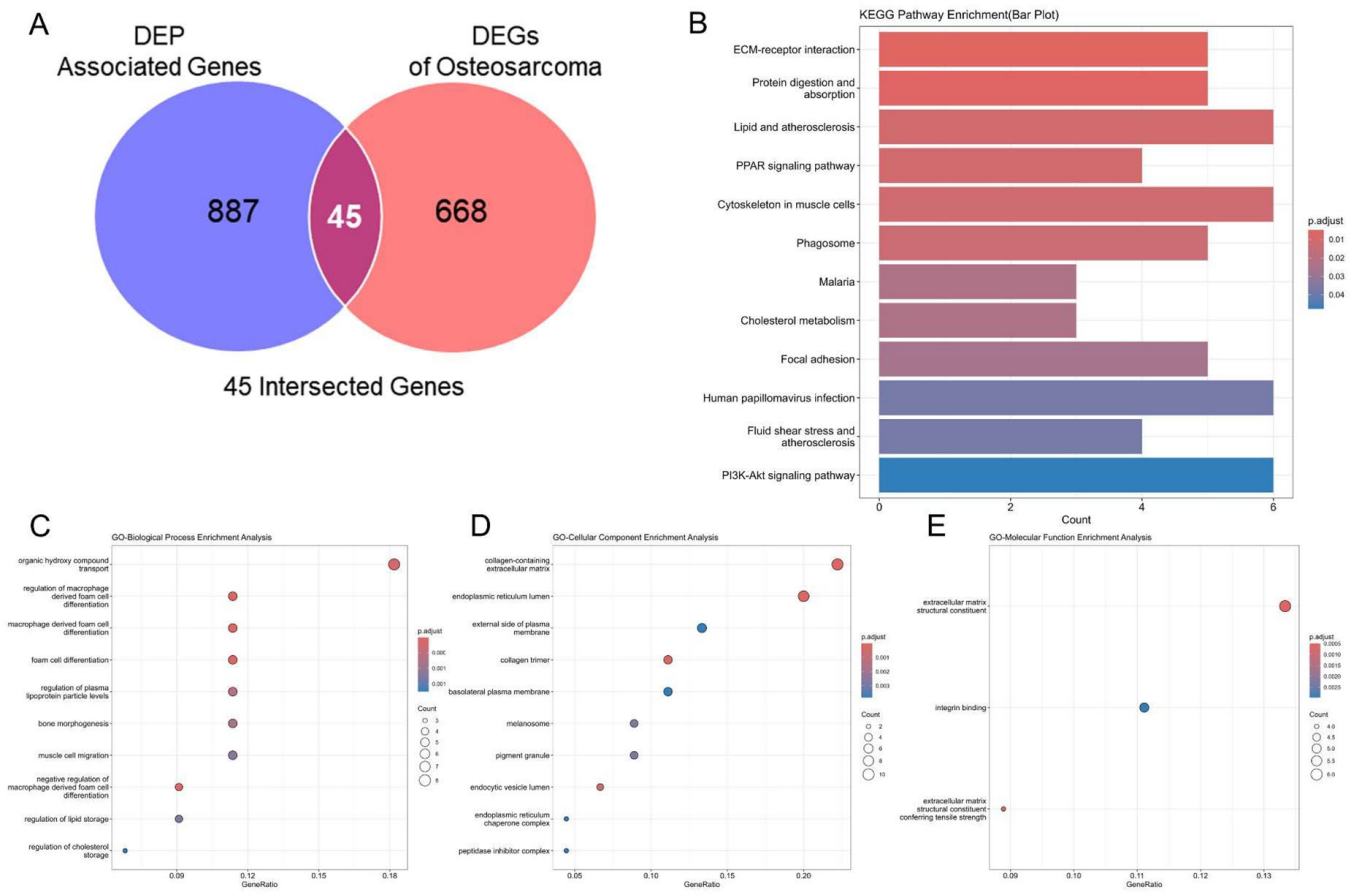
Integration of DEP-related genes and DEGs and functional enrichment. (A) Venn diagram displaying 45 overlapping genes between DEP-related targets and OS DEGs. (B) KEGG enrichment analysis of 45 overlapping genes. (C-E) GO enrichment analysis of Biological Process (BP), Cellular Component (CC) and Molecular Function (MF). Enrichment analysis was performed using the clusterProfiler package; terms with adjusted *p* < 0.05 were considered significant.

A total of 713 DEGs were intersected with 932 DEP-associated genes provided in Supplementary Table S2 curated from multiple toxicogenomic databases. This intersection yielded 45 overlapping genes were showed in Supplementary Table S3, which were considered putative DEP-responsive and OS-relevant genes (Figure 3A). To uncover the biological significance of these overlapping targets, we conducted KEGG and GO enrichment analyses. KEGG enrichment revealed that these 45 genes were significantly enriched in several cancer- and metabolism-related pathways. Notably, the most significantly enriched pathway was the ECM-receptor interaction (Figure 3B), which plays a pivotal role in tumour cell adhesion, invasion, and matrix remodelling^36^. Additional enriched pathways included the protein digestion and absorption, lipid and atherosclerosis, PPAR signalling pathway, and cytoskeleton in muscle cells (Figure 3B). For BP category, genes were enriched in organic hydroxy compound transport, foam cell differentiation, and bone morphogenesis, which may be associated with osteogenic activity and tumour metabolic adaptations (Figure 3C). In the CC category, the genes were enriched in collagen-containing ECM, endoplasmic reticulum lumen, and external side of the plasma membrane, suggesting involvement in extracellular remodelling and secretion (Figure 3D). In the MF category, top enriched terms included ECM structural constituent and integrin binding, indicating the genes’ roles in matrix organisation and cell-ECM interaction (Figure 3E). These enrichment results collectively indicate that the intersection between DEP targets and OS-DEGs may functionally converge on ECM remodelling, lipid regulation, and key oncogenic signalling axes-providing a biologically meaningful basis for subsequent hub gene prioritisation and validation.

### Construction of the PPI network and identification of hub nodes

**Figure 4.**
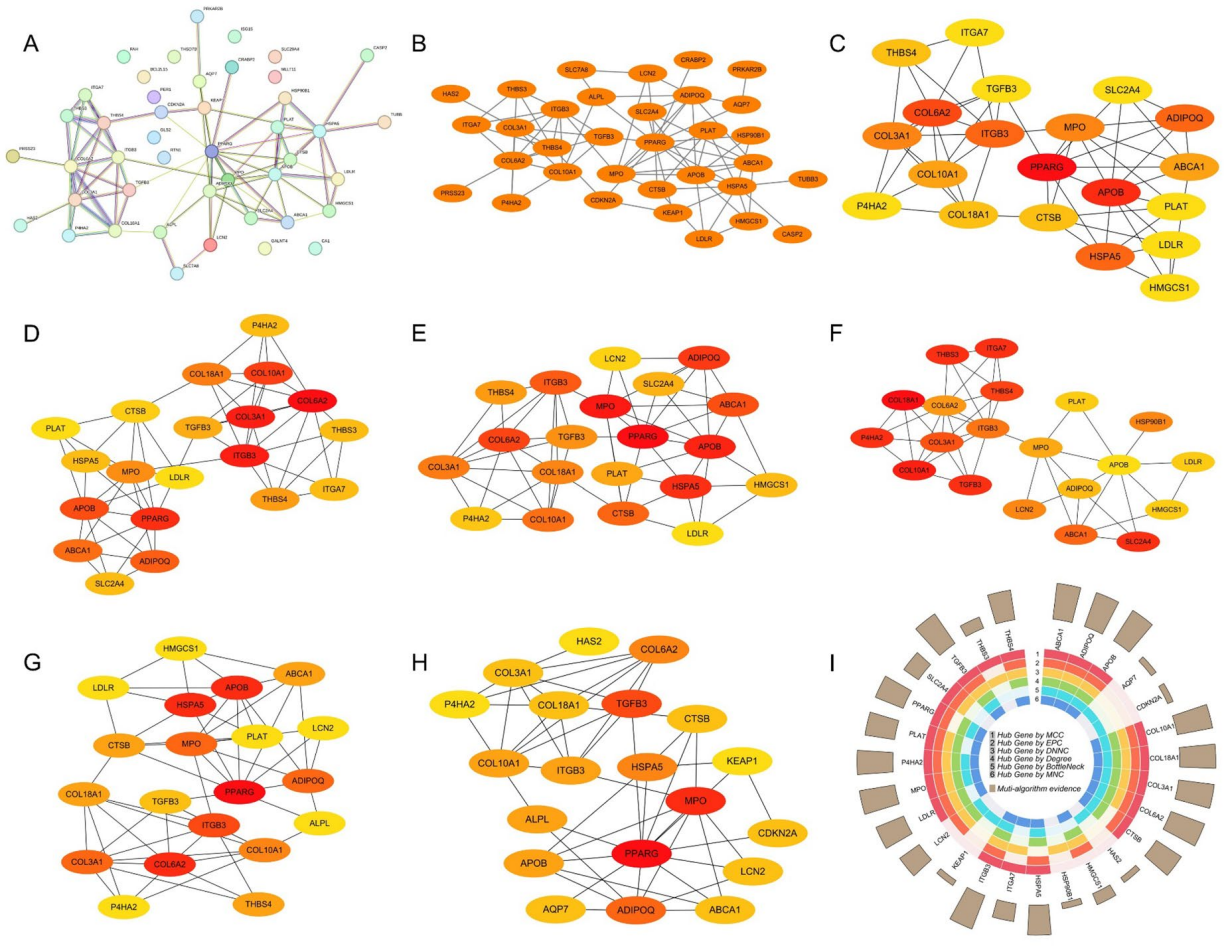
Construction and analysis of the PPI network from DEP and DEGs overlapping genes. (A) PPI network of the 45 overlapping genes generated using the STRING database. PPI enrichment *p*-value < 1.0e-16 indicates non-random biological connectivity. (B) Simplified network comprising 33 connected proteins after removing isolated nodes using Cytoscape. (C-H) Topological analysis using six cytoHubba algorithms (MCC, MNC, EPC, DMNC, Degree, BottleNeck) to rank centrality of nodes. (I) Venn diagram displaying 11 consistently prioritised hub genes identified across all six algorithms.

A PPI network was constructed using the STRING database to assess the functional connectivity among the 45 overlapping genes. The resulting network consisted of 44 nodes and 79 edges, with an average node degree of 3.59 and an average local clustering coefficient of 0.475. The network showed significant enrichment compared to random expectation (PPI enrichment *p* values < 1.0e-16), indicating substantial biological connectivity among these proteins (Figure 4A). The network data were then imported into Cytoscape software for visualisation and refinement. By removing isolated nodes, a simplified network comprising 33 interconnected proteins was obtained (Figure 4B). To prioritise key regulatory nodes within the network, we applied six topological algorithms implemented in the cytoHubba plugin, including MCC, MNC, EPC, DMNC, Degree, and BottleNeck (Figure 4C-H). The top 20 genes identified by each algorithm were intersected, resulting in 11 consistently prioritised hub proteins, ABCA1, ADIPOQ, APOB, COL10A1, COL18A1, COL3A1, COL6A2, ITGB3, MPO, P4HA2, and TGFB3 (Figure 4I).

### Feature selection using machine learning algorithms

**Figure 5.**
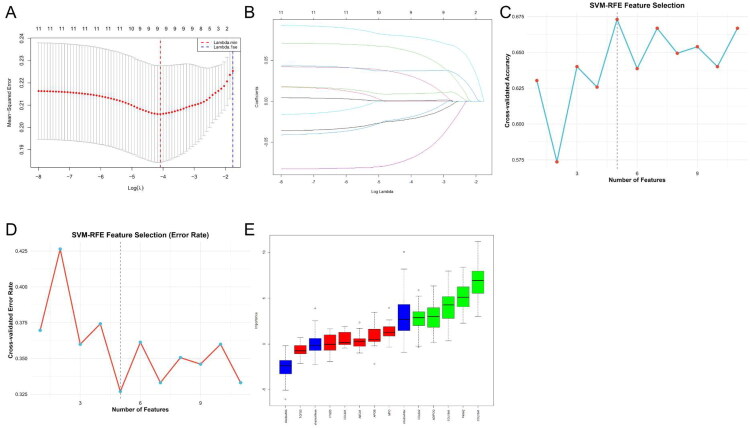
Machine learning based prioritisation of key hub genes from PPI network. (A, B) LASSO regression with 10-fold cross-validation identified five genes with optimal lambda minimising mean squared error (MSE). (C, D) Support Vector Machine Recursive Feature Elimination (SVM-RFE) using radial basis function kernel achieved highest accuracy with four genes; accuracy and error plotted versus number of features. (E) Boruta algorithm ranked feature importance using random forest classification, identifying six confirmed relevant genes.

Three machine learning algorithms, LASSO regression, SVM-RFE, and Boruta, were independently applied to the 11 hub genes to prioritise key molecular candidates. COL18A1, P4HA2, and COL10A1 emerged as robust predictors consistently identified across all models. LASSO regression was performed with 10-fold cross-validation to determine the optimal regularisation parameter (λ). A total of five key features were retained at the λ value minimising the mean squared error (MSE) (Figure 5A, B). Subsequently, SVM-RFE was conducted using a radial basis function (svmRadial) kernel, with 10-fold cross-validation employed to optimise the feature subset. The highest classification accuracy was achieved with four features, and both the accuracy and error rate trends were visualised across feature numbers (Figure 5C, D). In the Boruta analysis, a random forest-based iterative feature selection approach identified 5 genes as “Confirmed” important features, with their relative importance displayed in a ranked bar plot (Figure 5E). Intersecting the results of the three machine learning approaches, three hub genes-COL18A1, P4HA2 and COL10A1, were consistently selected. These genes were prioritised for subsequent molecular docking validation and scRNA-seq analysis.

### Single-cell transcriptomic landscape of OS reveals cell-type specific expression of DEP-associated hub genes

**Figure 6.**
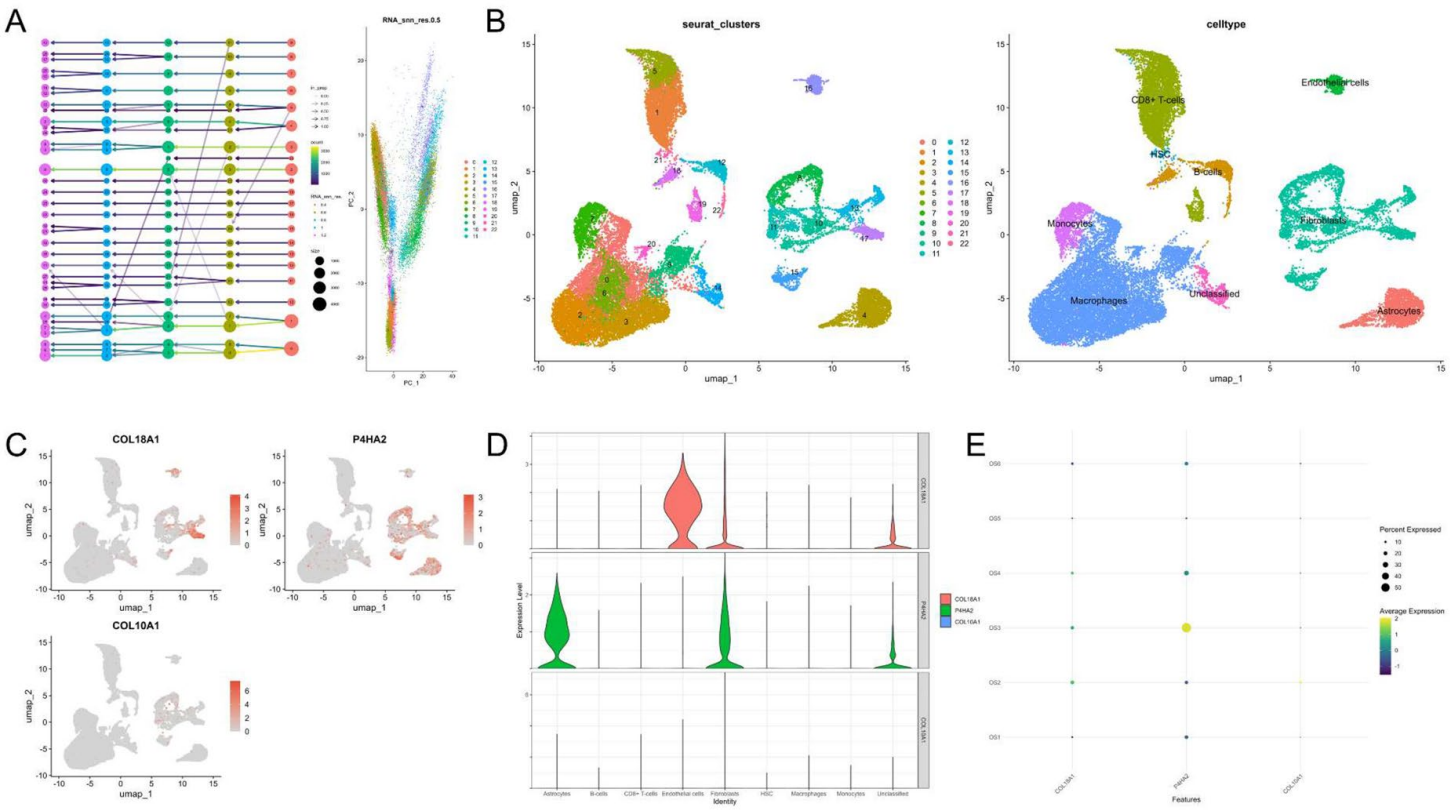
scRNA-seq analysis of DEP-associated genes in OS. (A) Clustree plot illustrating cluster stability across resolutions; resolution = 0.5 was selected for downstream analysis. (B) UMAP projection of 29,009 high-quality cells after Harmony-based batch correction, revealing 22 distinct clusters. Clusters were annotated into eight major cell types, including macrophages, CD8^+^ T cells, fibroblasts, endothelial cells, B cells, monocytes, astrocytes, and haematopoietic stem cells (HSCs), with some labelled as “unclassified.” (C) FeaturePlot visualisation showing spatial expression of three hub genes (COL18A1, P4HA2, COL10A1) across cell types. (D) Violin plots displaying the expression distributions of COL18A1, P4HA2, and COL10A1 among annotated cell types. (E) DotPlot summarising scaled expression levels and detection percentages of the hub genes in each cell population.

Analysis of six OS samples from the GSE162454 single-cell dataset revealed the spatial expression patterns of the three hub genes within the tumour microenvironment. After stringent quality control and batch effect correction, a total of 29,009 high-quality cells and 24,611 genes were retained for downstream analysis. We first identified the top 2,000 highly variable genes, followed by PCA, from which the top 15 principal components were selected for dimensionality reduction (Supplementary Figure S1) Harmony-based batch correction and graph-based clustering at resolution 0.5 yielded 22 distinct clusters, visualised via UMAP. A clustree plot further validated clustering consistency across multiple resolutions (Figure 6A). Using canonical lineage markers (Supplementary Figure S2), these clusters were manually annotated into eight major cell types: macrophages, CD8^+^ T cells, fibroblasts, endothelial cells, B cells, monocytes, astrocytes, and haematopoietic stem cells (HSCs) (Figure 6B). Some clusters remained unclassified. Marker gene expression was visualised using FeaturePlot, VlnPlot and DotPlot, highlighting distinct expression programs for each cluster. We then examined the expression patterns of three DEP-associated hub genes, COL18A1, P4HA2, and COL10A1. FeaturePlot analysis revealed that COL18A1 was predominantly enriched in endothelial cells and fibroblasts, whereas P4HA2 was highly expressed in fibroblasts and astrocytes (Figure 6C). In contrast, COL10A1 displayed low or negligible expression across all cell types (Figure 6C). These spatial trends were corroborated by violin and dot plots (Figure 6D, E), suggesting a cell type-specific role of COL18A1 and P4HA2 in remodelling the tumour stroma of OS.

### Molecular docking and molecular dynamics simulations reveals specific binding interactions between DEP and key protein targets

Molecular docking and molecular dynamics simulations were employed to elucidate the interactions between DEP-P4HA2 and DEP-COL18A1. The lowest-energy conformations identified from the energy landscape of the molecular simulations were selected for subsequent structural analyses.

**Figure 7.**
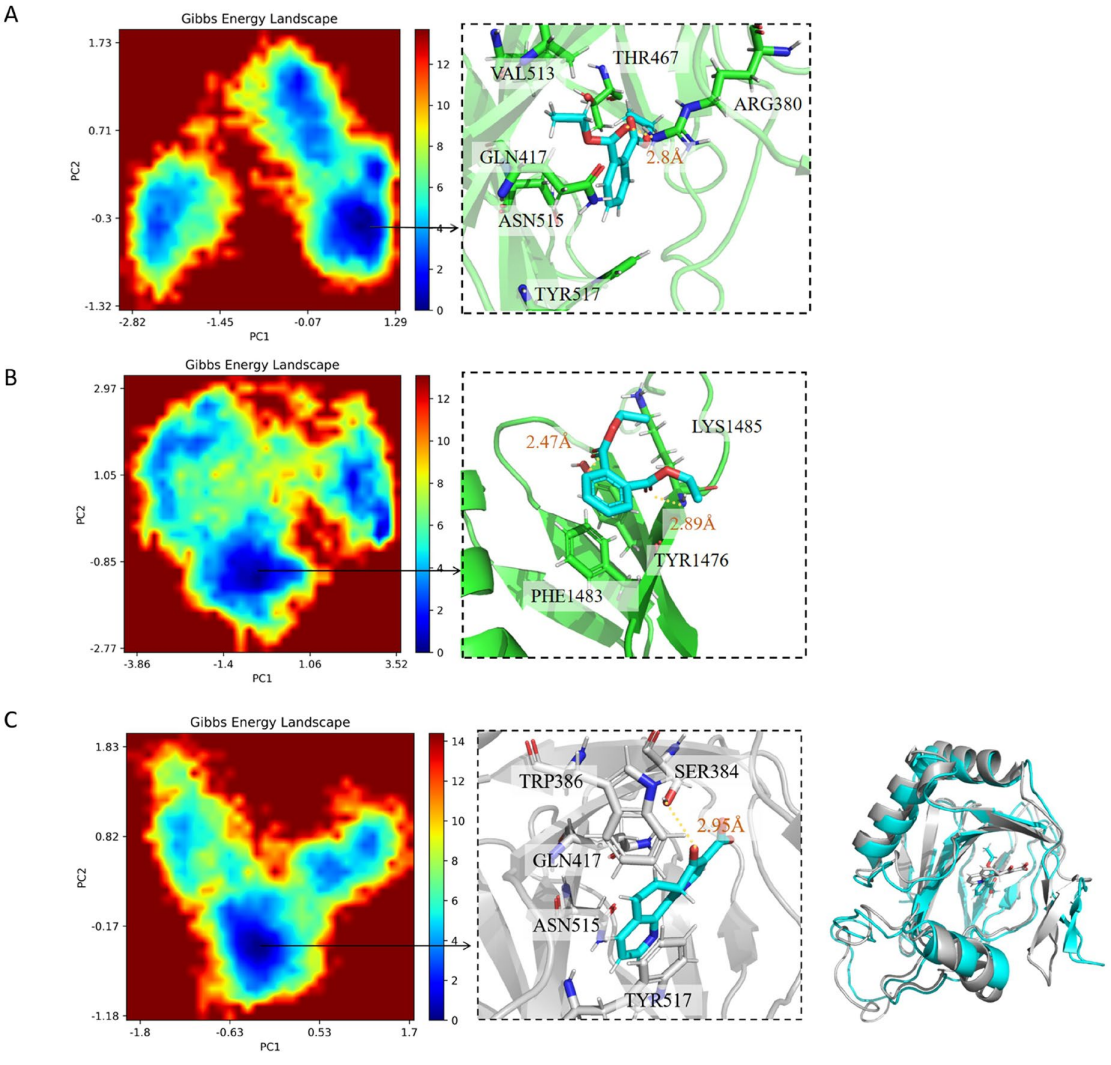
Energy landscapes and binding mode analyses of DEP-P4HA2, DEP-COL18A1, and 1,4-DPCA-P4HA2 complexes based on molecular dynamics simulations. (A) Left: the energy landscape of the DEP-P4HA2 complex MD trajectories; Right: the most stable conformation of the DEP-P4HA2 complex, highlighting key interaction residues. (B) Left: the energy landscape of the DEP-COL18A1 complex MD trajectories; Right: the most stable conformation of the DEP-COL18A1 complex, highlighting key interaction residues. (C) Left: the energy landscape of the 1,4-DPCA-P4HA2 complex MD trajectories; Middle: the most stable conformation of the DEP-COL18A1 complex, highlighting key interaction residues; Right: Structural superposition of the P4HA2-1,4-DPCA complex.

**Figure 8.**
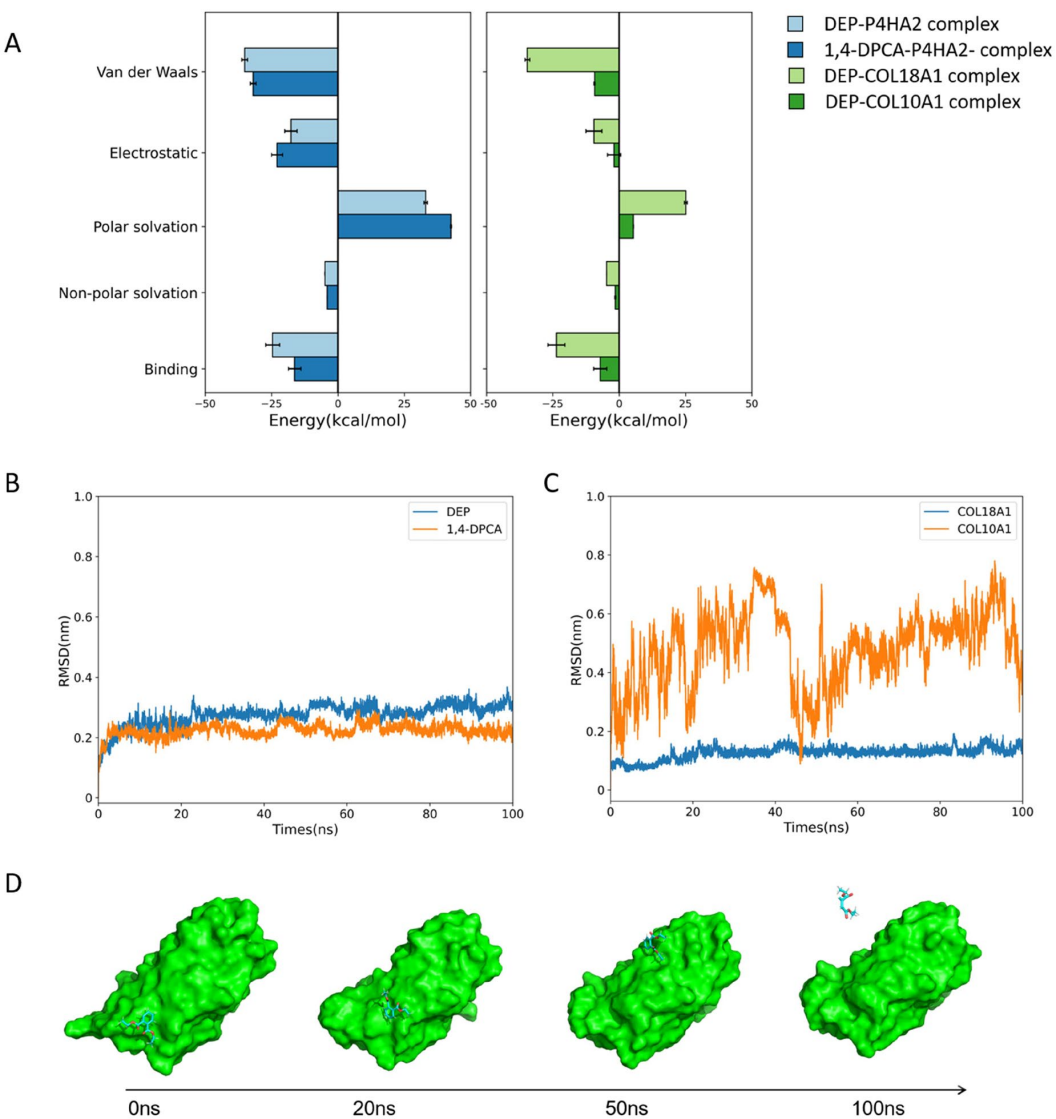
MM/PBSA binding free energy decomposition and RMSD analysis of DEP–protein complexes. (A) MM/PBSA calculations were performed to decompose the total binding free energy into Van der Waals, electrostatic, polar solvation, and non-polar solvation terms. Light green: Light blue: DEP-P4HA2; Dark blue:1,4-DPCA-P4HA2; Light Green: DEP-COL18A1; Dark green: DEP-COL10A1. The error bars indicate the standard deviation over the last 20 ns of the simulation. (B) RMSD plots of the backbone atoms over the 100 ns simulation trajectories for DEP-P4HA2 and 1,4-DPCA-P4HA2, indicating the structural stability of the complexes. (C) RMSD plots of the backbone atoms over the 100 ns simulation trajectories for DEP-COL18A1, and DEP-COL10A1, indicating the structural stability of the complexes. (D) Snapshot of the molecular dynamics simulation for the DEP-COL10A1 complex.

Regarding P4HA2, DEP binds primarily through a combination of hydrophobic interactions and hydrogen bonding. Residues GLN417, THR467, VAL513, ASN515, and TYR517 collectively form a hydrophobic microenvironment that stabilises the ligand conformation, while ARG380 establishes a hydrogen-bonding network with DEP, enhancing binding specificity (Figure 7A). Similarly, for the known inhibitor 1,4-DPCA, residues TRP386, GLN417, ASN515, and TYR517 delineate the binding pocket, providing steric and hydrophobic confinements to stabilise the ligand scaffold. In parallel, SER384 forms a robust hydrogen bond with the ligand, serving as a critical anchor to secure 1,4-DPCA within the active site (Figure 7C). MM/PBSA analysis estimated the binding free energy at −24.58 ± 2.65 kcal/mol—stronger than that of the known inhibitor 1,4-DPCA (17.26 ± 2.38 kcal/mol) (Figure 8A). Moreover, the 100 ns molecular dynamics simulation showed a stable RMSD profile for the DEP-P4HA2 complex, further confirming the robustness of this interaction (Figure 8B).

For COL18A1, stable hydrogen bonds were formed between DEP and residues TYR1476 and LYS1485 (Figure 7B). Notably, TYR1476 also contributed hydrophobic interactions, while the side chain of PHE1483 engaged in π-π stacking with DEP, further stabilising the complex conformation (Figure 7B). A 100 ns molecular dynamics simulation demonstrated that DEP maintains a stable interaction with COL18A1 throughout the trajectory. MM/PBSA calculations yielded a binding free energy of −23.57 ± 3.19 kcal/mol for the DEP-COL18A1 complex (Figure 8A). In contrast, the DEP-COL10A1 complex exhibited a significantly weaker binding affinity (−7.09 ± 2.47 kcal/mol) (Figure 8A), and RMSD analysis revealed substantial conformational fluctuations over the 100 ns simulation, suggesting either weak binding or lack of stable association (Figure 8C, 8D). Notably, this aligns with scRNA-seq data, where COL10A1 exhibited minimal expression, reinforcing its lower relevance in DEP-associated OS mechanisms.

Collectively, these findings provide structural evidence that DEP may exert biological effects in OS by directly interacting with both COL18A1 and P4HA2, potentially ECM composition and thereby contributing to tumour progression.

### Validation of hub gene expression and diagnostic accuracy

**Figure 9.**
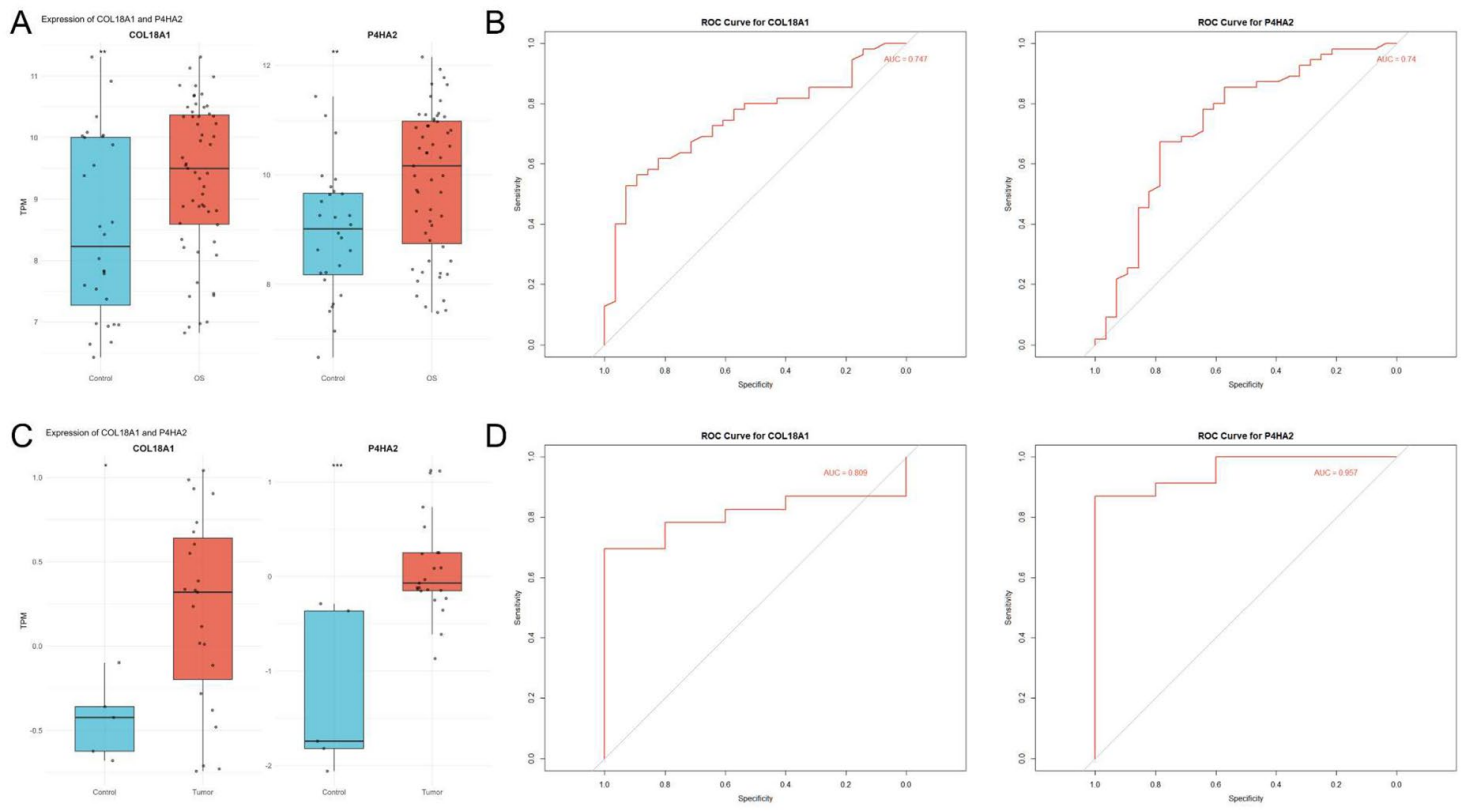
Validation of hub gene expression and diagnostic performance in GEO datasets. (A) Boxplots showing expression levels of COL18A1 and P4HA2 in the GEO training set. (B) ROC curves and AUC values of COL18A1 and P4HA2 in the training set. (C) Boxplots of gene expression in the independent validation cohort (GSE19276). (D) ROC curves of COL18A1 and P4HA2 in the validation set, showing high AUC values.

The diagnostic potential of P4HA2 (Figure 9A) and COL18A1 (Figure 9C) was rigorously evaluated across two independent transcriptomic cohorts. In the discovery cohort, both genes showed moderate classification accuracy (AUC up to 0.74), indicating initial diagnostic potential (Figure 9B). More compellingly, in the independent validation set (GSE19276), both genes achieved substantially higher AUCs (0.809 for COL18A1 and 0.957 for P4HA2) (Figure 9D), demonstrating robust reproducibility and strong discriminatory power. These findings underscore the reliability of COL18A1 and P4HA2 as candidate biomarkers for OS diagnosis, especially in the context of DEP-related molecular alterations.

## Discussion

In this study, we systematically identified and validated OS-associated key genes linked to DEP exposure by integrating multiple high-throughput transcriptomic datasets. A comprehensive multi-omics framework was developed, incorporating differential expression analysis, PPI network construction, machine learning algorithms, molecular docking, MD simulations, and single-cell transcriptomic validation. Initially, potential DEP target genes were obtained from several toxicogenomics databases, including TargetNet, ChEMBL, the CTD, and SwissTargetPrediction. These genes were then intersected with DEGs derived from OS transcriptomes. Network topological analysis using Cytoscape and various centrality algorithms helped prioritise hub candidates, which were further refined using LASSO regression, SVM-REF and Boruta. The stability and thermodynamic properties of the complexes between DEP and hub proteins were further verified through 100 ns MD simulations and MM/PBSA calculations. In parallel, the spatial distribution of these hub genes was examined within the tumour microenvironment using scRNA data. Finally, external validation was performed using the GEO datasets. Collectively, this integrative strategy offers multi-level evidence for the potential mechanisms by which environmental carcinogens may contribute to OS pathogenesis and provides a conceptual framework for identifying novel therapeutic targets.

DEP is a prevalent environmental contaminant widely used in plastic materials, cosmetics, and medical devices. Chronic DEP exposure has been linked to various health risks and is increasingly implicated in oncogenesis^1^. However, current research on DEP’s role in tumorigenesis remains limited, primarily focusing on hormone-related malignancies such as breast cancer ^11^^,^^37^, and lacks a clear understanding of its mechanistic pathways. Emerging studies suggest that DEP may exert toxic effects by inducing oxidative stress, mitochondrial damage, and apoptosis^38^. DEP exposure has also demonstrated developmental toxicity, particularly affecting neuroectodermal cells, with markers such as Pax6, Nestin, Sox1, and Sox3 significantly upregulated post-exposure^39^. In this study, we used network toxicology, molecular docking technology, and single-cell sequencing to explore the relationship between DEP and OS.

In the present study, by integrating toxicogenomic databases and OS specific transcriptomic data, we identified 45 potential DEP-interacting genes. Our enrichment analysis of these genes revealed significant involvement in several hallmark pathways of tumour progression, offering compelling evidence that DEP may influence OS through multifactorial mechanisms. In the KEGG pathway enrichment, the ECM-receptor interaction pathway demonstrated the most significant enrichment. This pathway is known to govern essential oncogenic processes such as tumour cell detachment, adhesion, matrix degradation, and proliferation^40^^,^^41^. It plays a pivotal role in the invasive potential of bone tumours such as OS and Ewing sarcoma^42^^,^^43^. Persistent activation of ECM-receptor signalling has been reported to promote cellular migration and metastasis. Additional enrichment of pathways including protein digestion and absorption, lipid and atherosclerosis, and the PPAR signalling pathway suggests that DEP may modulate tumour progression by altering the metabolic landscape of the tumour microenvironment, particularly through lipid handling and extracellular nutrient processing. From the MF perspective, key terms such as ECM structural constituent and integrin binding were prominently enriched. This implies that DEP-related targets may regulate collagen synthesis or degradation, thus altering the adhesive and migratory capacities of tumour cells via remodelling of ECM integrity and integrin signalling^44^^,^^45^. In the CC category, genes were primarily enriched in collagen-containing ECM, endoplasmic reticulum lumen, and external side of plasma membrane, indicating roles in extracellular remodelling, protein secretion, and stress adaptation. Regarding BP category, notable enrichment was observed in foam cell differentiation, lipid storage and transport, bone morphogenesis, and muscle cell migration. These findings are consistent with the mesenchymal origin and plasticity of OS cells and suggest potential links between DEP and immune-metabolic modulation of tumour behaviour^46^. Collectively, GO and KEGG enrichment analyses highlight that DEP may orchestrate OS progression through ECM remodelling, metabolic reprogramming, signal transduction, and immune regulation.

To explore the potential molecular mediators of DEP-induced OS progression, we first constructed a PPI network and applied multiple topological algorithms using Cytoscape to prioritise key nodes. Subsequently, we employed three widely recognised machine learning algorithms, LASSO regression, SVM-RFE, and Bourta, for integrative feature selection. Through intersecting the outputs of all three models, we identified three hub candidates, P4HA2, COL18A1, and COL10A1. Initial validation revealed that P4HA2 and COL18A1 were consistently selected across methods and exhibited robust expression patterns. In contrast, COL10A1, although algorithmically selected, displayed weak or absent expression in scRNA-seq data, and no significant binding affinity in molecular docking. Therefore, COL10A1 was logically excluded, ensuring the scientific rigour of our hub gene selection.

In the tumour microenvironment OS, ECM remodelling and aberrant angiogenesis are critical drivers of malignancy progression^47–49^. Through a comprehensive multi-omics approach, we identified two DEP-associated genes, P4HA2 and COL18A1, which were further validated in terms of function, mechanism, spatial expression, and clinical relevance. P4HA2, a subtype of prolyl 4-hydroxylase, catalyses the hydroxylation of proline residues in collagen, thereby stabilising the triple-helical structure. Besides collagen, other P4HA2 substrates may include collagen-like proteins such as apoproteins^50^ and Argonaute 2^51^. Overexpression of P4HA2 promotes ECM deposition and enhances tumour invasiveness, particularly under hypoxic conditions^52^^,^^53^. Although understudied in OS, recent evidence suggests its involvement in OS progression via MAPK signalling^54^. Our single-cell analysis revealed that P4HA2 is mainly expressed in fibroblasts and astrocyte-like cells, supporting its role in ECM regulation. COL18A1, encoding the α-chain of collagen XVIII, is a major component of vascular basement membrane. Its C-terminal fragment, endostatin, is a potent angiogenesis regulator with dual functionality^55^. In breast cancer, COL18A1 is known to sustain cancer stemness via ErbB signalling and promote tumour progression^56^. Our scRNA results show high expression of COL18A1 in endothelial and fibroblast populations, indicating its involvement in vascular dynamics and stromal remodelling. Notably, previous studies have suggested that tumour-associated fibroblasts may release bioactive COL18A1 fragments to facilitate metastasis^57^. To further valid the stability of DEP-protein interactions, we performed 100-ns MD simulations and MM/PBSA binding free energy calculations. The results revealed that DEP forms stable complexes with P4HA2 and COL18A1 through multiple interaction modes beyond simple hydrogen bonding. For P4HA2, DEP is stabilised by a hydrophobic microenvironment (formed by residues such as GLN417, VAL513, and TYR517) and a hydrogen-bond network with ARG380. Similarly, the DEP-COL18A1 complex is maintained by hydrogen bonds involving TYR1476, and LYS1485 as well as π-π stacking interactions with PHE1483. MM/PBSA calculations indicated favourable binding free energies for both P4HA2 (ΔG = −24.58 ± 2.65 kcal/mol) and COL18A1 (ΔG = −23.57 ± 3.19 kcal/mol), whereas COL10A1 exhibited weaker and unstable binding. These computational findings provide a structural basis for the hypothesis that DEP may potentially modulate the catalytic activity or stability of these ECM-modifying enzymes, thereby influencing the tumour microenvironment. The diagnostic potential of COL18A1 and P4HA2 was rigorously evaluated across two independent transcriptomic cohorts. In the discovery cohort, both genes showed moderate classification accuracy (AUC up to 0.71), indicating initial diagnostic potential. More compellingly, in the independent validation set (GSE19276), both genes achieved substantially higher AUCs, demonstrating robust reproducibility and strong discriminatory power. Collectively, these findings support a conceptual model in which DEP exposure activates specific molecular targets, leading to remodelling of the tumour microenvironment and promoting OS progression. This mechanistic framework underscores the potential of targeting DEP-responsive pathways for therapeutic intervention.

In summary, by integrating differential gene expression analysis, functional enrichment, protein-protein interaction networks, machine learning algorithms, molecular docking and MD simulations, single-cell RNA-sequencing, and external dataset validation, this study systematically identified P4HA2 and COL18A1 as key DEP-responsive genes potentially involved in OS progression. Through multi-layered bioinformatic analyses, we constructed a transcriptomic regulatory landscape revealing how DEP may reshape the tumour microenvironment by modulating ECM remodelling, angiogenesis, and signal transduction pathways. Importantly, molecular docking, MD simulations and scRNA expression analyses suggest that DEP may directly bind to specific protein targets within defined cell subpopulations, thereby facilitating malignancy through precise microenvironmental remodelling. These findings provide novel mechanistic insights into how environmental toxicants such as DEP contribute to tumorigenesis and highlight promising targets for future diagnostic or therapeutic strategies.

## Conclusion

This study provides the first comprehensive insight into the potential oncogenic mechanisms of DEP in OS. Through high-throughput multi-omics analysis, we identified P4HA2 and COL18A1 as DEP-associated key regulators with strong binding affinity, elevated expression in OS, and robust diagnostic potential. These findings enhance our understanding of the link between environmental exposure to DEP and tumorigenesis and lay a solid molecular foundation for future therapeutic targeting strategies in OS.

## Supplementary Material

Maked up_Supplementary Material.docx

## Data Availability

All data used in this study are publicly available. Gene expression datasets were obtained from the Gene Expression Omnibus (GEO) database under accession numbers GSE99671, GSE39058, GSE19276, and GSE162454. Toxicogenomic data were retrieved from public resources including CTD (http://ctdbase.org/), ChEMBL (https://www.ebi.ac.uk/chembl/), SwissTargetPrediction (http://www.swisstargetprediction.ch/), and TargetNet (http://targetnet.scbdd.com/).
