# Supplementary material for: Diethyl Phthalate (DEP) as a potential osteosarcoma risk factor: a multi-omics study integrating network Toxicology, single-cell RNA sequencing, and molecular docking
Source: J Enzyme Inhib Med Chem. 2026 Feb 16;41(1):2611582. doi: 10.1080/14756366.2025.2611582 (PMC12912210; doi:10.1080/14756366.2025.2611582)
Supplement: Maked up_Supplementary Material.docx [file IENZ_A_2611582_SM0725.docx]

**Supplementary Material**

**Diethyl Phthalate (DEP) as a Potential Osteosarcoma Risk Factor: A Multi-Omics Study Integrating Network Toxicology, Single-cell RNA sequencing, and Molecular Docking**

**
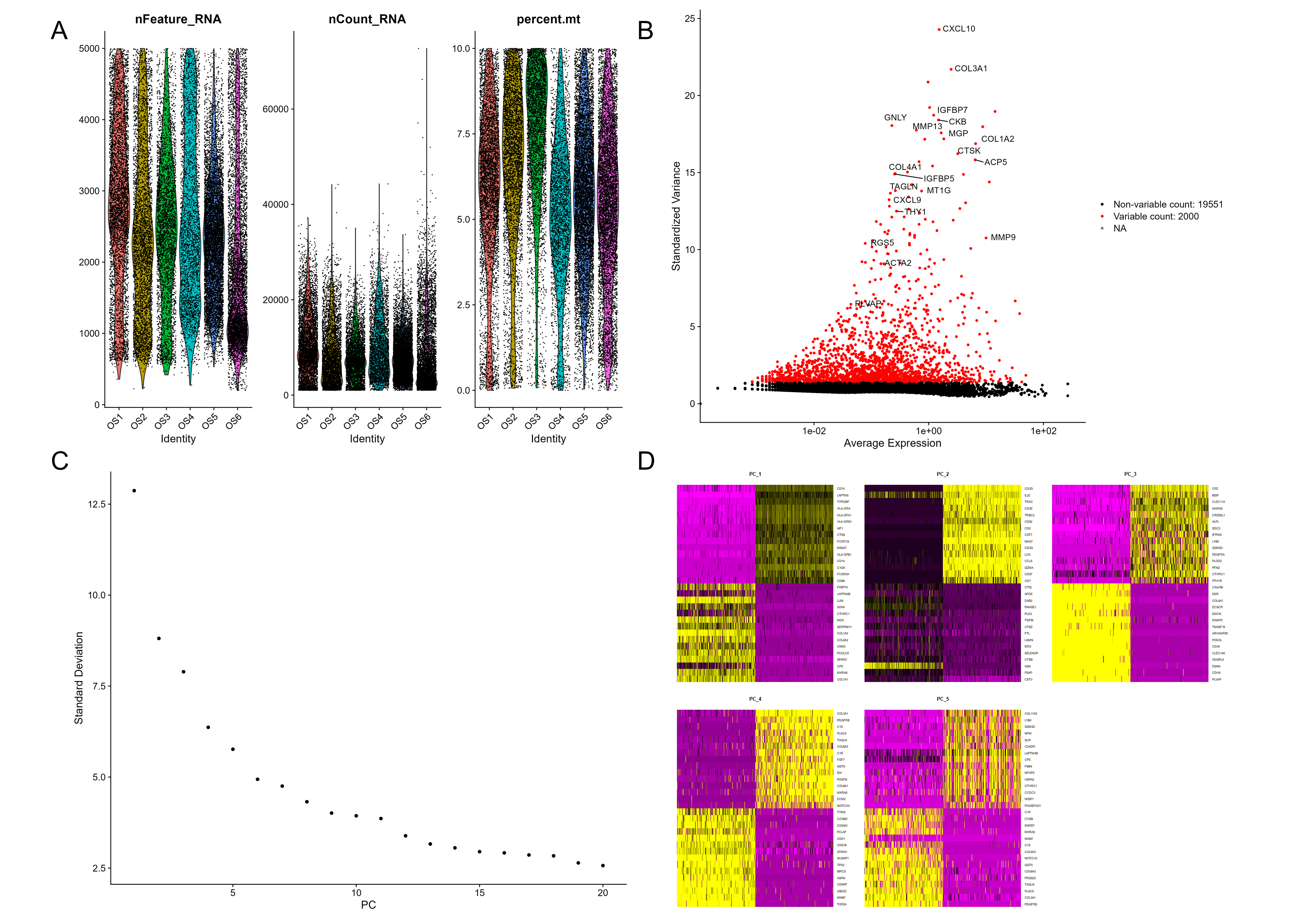
**

**FigureS1.** Quality control and differential expression analysis of GSE162454 scRNA-seq data in OS patients.

(A) Violin plots showing the distribution of total features (nFeature_RNA), total counts (nCount_RNA), and mitochondrial gene percentage (percent.mt) across all samples. These parameters were used to filter out low-quality cells. (B) Scatter plot displaying the top 2,000 highly variable genes (HVGs), highlighting the top 20 most variable genes ranked by average dispersion. These genes were selected to enhance sensitivity in capturing cell-to-cell transcriptional heterogeneity during PCA and clustering. (C) Elbow plot showing the standard deviation of each of the top 50 principal components. (D) PCA heatmap visualization of the top 5 principal components. Each plot displays gene loadings and variance explained, reflecting the ability of these components to capture biologically meaningful variation among cells prior to clustering.

**
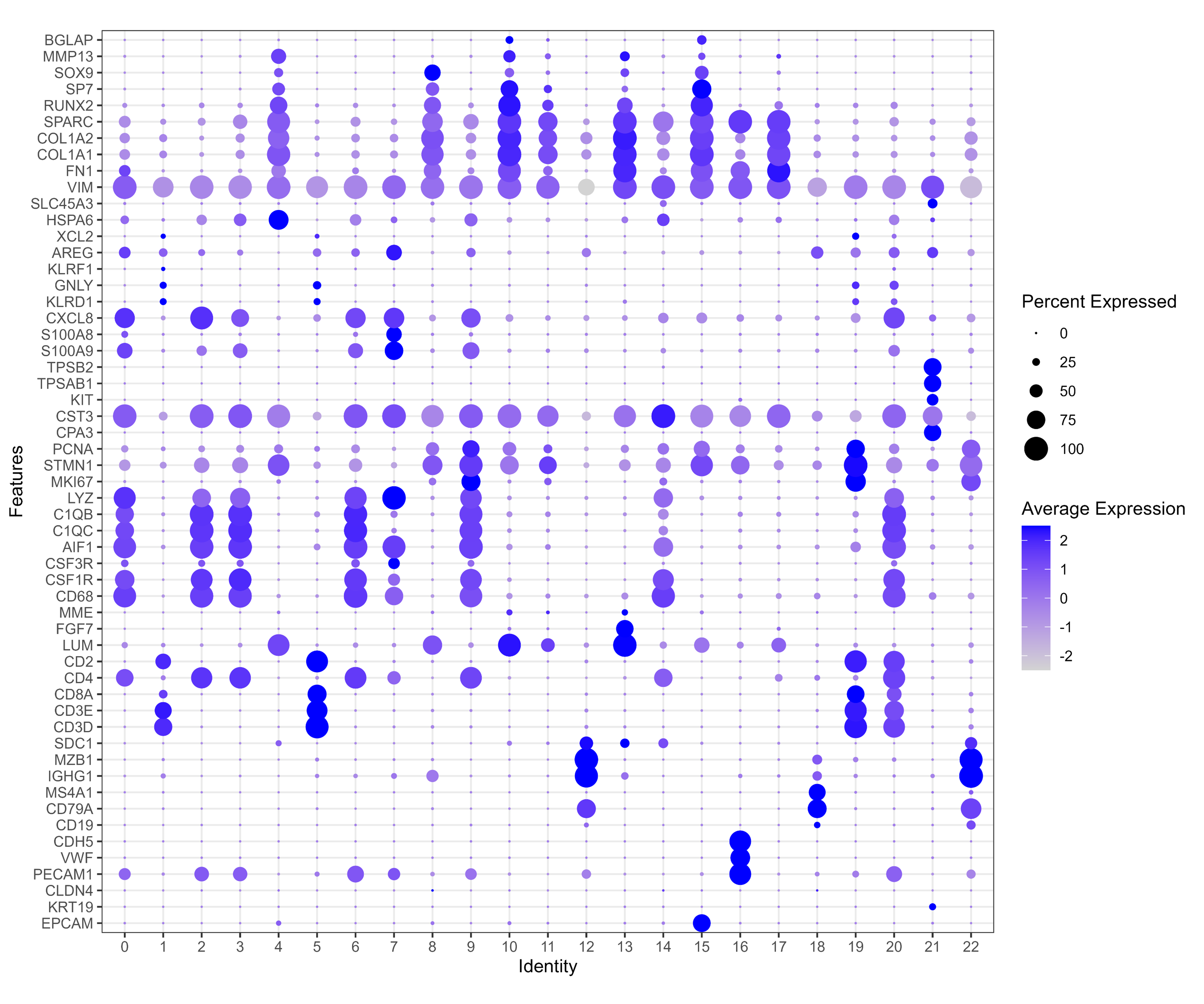
**

**FigureS2.** Canonical lineage marker expression in scRNA-seq of osteosarcoma samples.

**TableS1.** Differentially expressed genes (DEGs) between osteosarcoma and control groups.

|  | **logFC** | **AveExpr** | **t** | **P.Value** | **B** |
| --- | --- | --- | --- | --- | --- |
| TMEM71 | -0.96949 | 6.917348 | -4.50133 | 2.08E-05 | 2.252822 |
| LCN2 | -1.19526 | 7.043206 | -4.32473 | 4.06E-05 | 1.712148 |
| YARS | 0.72433 | 10.40695 | 4.261328 | 5.14E-05 | 1.521145 |
| HAS2 | 1.086805 | 8.175105 | 4.224209 | 5.89E-05 | 1.410099 |
| ALAS2 | -1.89165 | 8.211977 | -4.11428 | 8.80E-05 | 1.08473 |
| HERC1 | -1.13246 | 9.988068 | -4.09836 | 9.32E-05 | 1.038051 |
| GNRH1 | -0.87345 | 6.894733 | -4.09115 | 9.57E-05 | 1.016946 |
| HEMGN | -1.27016 | 7.923569 | -4.0879 | 9.68E-05 | 1.007433 |
| ANO2 | -0.62815 | 6.63974 | -4.01373 | 0.000126 | 0.791793 |
| PLOD1 | 0.697473 | 10.92381 | 3.997375 | 0.000134 | 0.744591 |
| EIF4G1 | 0.883923 | 11.21275 | 3.996246 | 0.000134 | 0.741338 |
| MMP8 | -0.99815 | 7.379849 | -3.97531 | 0.000145 | 0.681099 |
| P4HA2 | -1.2779 | 6.472738 | -3.96998 | 0.000148 | 0.665777 |
| AKR1A1 | 0.789649 | 8.457593 | 3.953195 | 0.000157 | 0.617682 |
| LEPRE1 | 1.026844 | 9.802416 | 3.936391 | 0.000166 | 0.569652 |
| CCL14 | -0.81014 | 8.093246 | -3.90441 | 0.000186 | 0.47862 |
| FAIM3 | -0.71999 | 7.201763 | -3.86981 | 0.00021 | 0.380669 |
| AMY2B | -0.67866 | 7.016601 | -3.86253 | 0.000216 | 0.360132 |
| EBF3 | -0.83205 | 8.188486 | -3.85908 | 0.000218 | 0.350413 |
| KIAA0182 | -0.52291 | 8.487921 | -3.84031 | 0.000233 | 0.29761 |
| COL18A1 | 1.430674 | 9.47064 | 3.823812 | 0.000247 | 0.251342 |
| FRMD3 | -0.64804 | 7.129538 | -3.82122 | 0.000249 | 0.244078 |
| YPEL1 | -0.85008 | 7.937094 | -3.81467 | 0.000255 | 0.225758 |
| STK3 | 0.945395 | 10.33188 | 3.782057 | 0.000285 | 0.134846 |
| HIF1AN | -0.56097 | 8.086092 | -3.77323 | 0.000294 | 0.110323 |
| BCO2 | -0.77239 | 6.314898 | -3.76778 | 0.000299 | 0.095203 |
| LOC338799 | -0.74013 | 6.277887 | -3.76665 | 0.0003 | 0.092077 |
| TXLNA | 0.763621 | 9.867261 | 3.741092 | 0.000328 | 0.021392 |
| RPH3A | -0.60906 | 6.528067 | -3.73511 | 0.000335 | 0.004887 |
| DDOST | 0.632859 | 9.69866 | 3.734598 | 0.000335 | 0.003484 |
| AOX2P | -0.58772 | 6.007206 | -3.73303 | 0.000337 | -0.00083 |
| FAM65B | -0.58844 | 7.569617 | -3.73047 | 0.00034 | -0.00789 |
| ATP1A2 | -1.07051 | 6.98194 | -3.72188 | 0.00035 | -0.03152 |
| THBS4 | -0.83588 | 7.62093 | -3.71156 | 0.000363 | -0.05988 |
| TAL1 | -0.64315 | 7.615484 | -3.69159 | 0.000388 | -0.11457 |
| HM13 | 0.819542 | 8.802557 | 3.688404 | 0.000393 | -0.12329 |
| THSD7B | -0.51054 | 6.538449 | -3.68506 | 0.000397 | -0.13243 |
| C1orf85 | 1.062426 | 9.154351 | 3.679881 | 0.000404 | -0.14656 |
| NPBWR2 | -0.59051 | 5.185285 | -3.67693 | 0.000408 | -0.15462 |
| HBA1 | -0.72185 | 6.437487 | -3.65842 | 0.000434 | -0.20499 |
| PARD3B | -0.59777 | 7.230544 | -3.65713 | 0.000436 | -0.2085 |
| KCNRG | -0.56958 | 7.64304 | -3.64668 | 0.000452 | -0.23686 |
| DYRK1B | -0.70896 | 6.235601 | -3.6237 | 0.000488 | -0.29902 |
| AMICA1 | -0.58936 | 7.712708 | -3.59626 | 0.000535 | -0.37289 |
| ITGA7 | -0.55305 | 6.642418 | -3.57584 | 0.000573 | -0.42761 |
| ABCA10 | -0.68885 | 7.178 | -3.5654 | 0.000593 | -0.45551 |
| COL10A1 | 0.782825 | 8.074814 | 3.550145 | 0.000624 | -0.49616 |
| UNC93A | -0.71434 | 5.509991 | -3.50461 | 0.000725 | -0.61677 |
| CDH19 | -0.63401 | 6.194267 | -3.48373 | 0.000777 | -0.67169 |
| SCN7A | -0.65668 | 6.718541 | -3.47071 | 0.000811 | -0.70584 |
| COL11A1 | 0.907401 | 11.5551 | 3.465379 | 0.000825 | -0.71978 |
| NFIA | -0.58759 | 8.864477 | -3.45344 | 0.000858 | -0.75097 |
| KLF15 | -0.57679 | 6.311912 | -3.4465 | 0.000877 | -0.76906 |
| MUL1 | 0.735723 | 8.562442 | 3.437477 | 0.000903 | -0.79253 |
| SDF4 | 0.897758 | 8.64816 | 3.435981 | 0.000908 | -0.79642 |
| ZBTB39 | -0.52523 | 8.24329 | -3.43574 | 0.000908 | -0.79705 |
| SERPINH1 | 0.797447 | 10.09683 | 3.428938 | 0.000929 | -0.81472 |
| NUP62 | 0.729089 | 9.912211 | 3.42682 | 0.000935 | -0.82021 |
| ABCA9 | -0.63815 | 7.221101 | -3.40894 | 0.000991 | -0.8665 |
| CD276 | 0.932678 | 9.034271 | 3.401759 | 0.001014 | -0.88505 |
| NFIB | -1.04065 | 9.96086 | -3.38895 | 0.001057 | -0.91806 |
| LENG9 | -0.77728 | 7.202899 | -3.38574 | 0.001068 | -0.9263 |
| ATP8A1 | -0.92402 | 8.279179 | -3.38551 | 0.001068 | -0.9269 |
| ACOT7 | 0.65949 | 8.20563 | 3.38486 | 0.001071 | -0.92857 |
| SOX10 | -0.54129 | 5.919851 | -3.38301 | 0.001077 | -0.93333 |
| TOMM34 | 0.762685 | 8.906256 | 3.379128 | 0.00109 | -0.9433 |
| GABRG3 | -0.54134 | 5.571862 | -3.37809 | 0.001094 | -0.94596 |
| KIAA0090 | 0.771976 | 10.26719 | 3.375506 | 0.001103 | -0.9526 |
| PCDHA1 | -0.6428 | 6.708484 | -3.3651 | 0.001141 | -0.97925 |
| AP1B1 | 0.799138 | 9.288547 | 3.359167 | 0.001162 | -0.99444 |
| CHL1 | -0.6212 | 7.664579 | -3.35241 | 0.001188 | -1.01171 |
| YIPF3 | 0.736699 | 10.67279 | 3.344298 | 0.001219 | -1.0324 |
| HBD | -1.09628 | 7.825956 | -3.34336 | 0.001223 | -1.03478 |
| RDH11 | 0.560424 | 10.61409 | 3.339055 | 0.00124 | -1.04575 |
| RPN2 | 0.56834 | 11.64946 | 3.336709 | 0.001249 | -1.05172 |
| NOC2L | 0.691544 | 7.87477 | 3.324543 | 0.001298 | -1.08262 |
| PCDHAC1 | -0.54468 | 6.746928 | -3.31078 | 0.001356 | -1.11748 |
| FAM55A | -0.82295 | 5.908975 | -3.30976 | 0.001361 | -1.12007 |
| MRPL52 | 0.881202 | 9.216281 | 3.299505 | 0.001405 | -1.14597 |
| MFAP2 | 1.31926 | 8.037576 | 3.296043 | 0.001421 | -1.1547 |
| C1orf144 | 0.84563 | 10.44337 | 3.292964 | 0.001435 | -1.16246 |
| ARG1 | -0.74562 | 6.533888 | -3.29271 | 0.001436 | -1.1631 |
| HSPA8 | 0.761229 | 12.31988 | 3.280162 | 0.001494 | -1.19466 |
| PLOD3 | 1.099157 | 9.545248 | 3.278419 | 0.001502 | -1.19904 |
| RAG2 | -0.76304 | 6.370527 | -3.26311 | 0.001576 | -1.2374 |
| PANX3 | 1.545618 | 9.334507 | 3.254257 | 0.001621 | -1.25954 |
| DSE | 0.661242 | 8.563758 | 3.253814 | 0.001623 | -1.26065 |
| NME2 | 0.628945 | 10.2054 | 3.250528 | 0.00164 | -1.26885 |
| BMP5 | -0.7458 | 7.458738 | -3.24909 | 0.001647 | -1.27244 |
| GPR180 | 0.771068 | 9.769944 | 3.248115 | 0.001652 | -1.27487 |
| PMP2 | -0.52367 | 6.248911 | -3.24627 | 0.001662 | -1.27946 |
| FAM189B | 1.061062 | 8.986554 | 3.243397 | 0.001677 | -1.28662 |
| BEST3 | 0.93951 | 9.57 | 3.237273 | 0.001709 | -1.30187 |
| PIP5K1B | -0.80619 | 7.237882 | -3.23704 | 0.00171 | -1.30244 |
| GNMT | -0.53604 | 5.093419 | -3.23652 | 0.001713 | -1.30374 |
| PIK3R2 | 0.908517 | 8.728275 | 3.232335 | 0.001736 | -1.31414 |
| FGF9 | -0.68238 | 6.883865 | -3.22948 | 0.001751 | -1.32123 |
| IFI6 | 0.574755 | 11.06493 | 3.225373 | 0.001774 | -1.33142 |
| HBA2 | -0.99702 | 10.90965 | -3.22472 | 0.001777 | -1.33305 |
| CALCOCO1 | -0.78594 | 9.097333 | -3.21897 | 0.001809 | -1.3473 |
| SLC30A7 | 0.568864 | 11.01352 | 3.198506 | 0.001928 | -1.39785 |
| SDR39U1 | -0.56142 | 7.042261 | -3.18503 | 0.00201 | -1.43102 |
| FCGBP | 1.317907 | 9.606991 | 3.176544 | 0.002064 | -1.45184 |
| CHMP4C | -0.55328 | 7.014534 | -3.17328 | 0.002084 | -1.45983 |
| HBG2 | -1.33627 | 6.892355 | -3.17255 | 0.002089 | -1.46162 |
| DIABLO | 0.550604 | 9.908872 | 3.166866 | 0.002126 | -1.47554 |
| UCK2 | 0.710649 | 7.604273 | 3.166189 | 0.00213 | -1.47719 |
| XK | -0.95265 | 6.995254 | -3.16476 | 0.00214 | -1.4807 |
| KAT2B | -0.64299 | 8.506486 | -3.16108 | 0.002164 | -1.48968 |
| ZADH2 | -0.7717 | 8.427853 | -3.15986 | 0.002172 | -1.49266 |
| C19orf53 | 0.514031 | 11.00218 | 3.157276 | 0.00219 | -1.49897 |
| FREM1 | -0.51912 | 6.68898 | -3.15229 | 0.002223 | -1.51112 |
| MMP25 | -0.56117 | 6.519164 | -3.1422 | 0.002293 | -1.53568 |
| HBB | -1.22953 | 11.76153 | -3.14081 | 0.002303 | -1.53907 |
| TUBA8 | -0.59904 | 6.553705 | -3.13764 | 0.002326 | -1.54677 |
| ABHD12 | 0.7446 | 7.688713 | 3.13322 | 0.002357 | -1.55751 |
| KEAP1 | 0.965826 | 8.920907 | 3.131858 | 0.002367 | -1.56081 |
| ERV3 | -0.61827 | 7.925226 | -3.13157 | 0.002369 | -1.56151 |
| PRG2 | -0.96908 | 7.432629 | -3.12384 | 0.002426 | -1.58024 |
| VAT1L | -0.67486 | 7.150257 | -3.11024 | 0.002528 | -1.61309 |
| C9orf84 | -0.59129 | 6.870058 | -3.10017 | 0.002607 | -1.63737 |
| BTLA | -0.51096 | 6.80917 | -3.09956 | 0.002612 | -1.63884 |
| PLAT | -0.81399 | 8.034698 | -3.09859 | 0.002619 | -1.64116 |
| KIAA0355 | -0.57087 | 8.473881 | -3.09591 | 0.002641 | -1.64761 |
| RIMKLB | -0.77038 | 8.812985 | -3.09228 | 0.00267 | -1.65634 |
| TRMT12 | 0.789443 | 8.408536 | 3.084746 | 0.002732 | -1.67441 |
| TRIM10 | -0.96984 | 6.678916 | -3.08214 | 0.002753 | -1.68067 |
| VSTM1 | -0.67958 | 5.929589 | -3.07381 | 0.002824 | -1.7006 |
| FAM98A | 0.531997 | 11.77755 | 3.072414 | 0.002835 | -1.70393 |
| CXCL14 | 0.943862 | 10.12096 | 3.070571 | 0.002851 | -1.70833 |
| FKBP14 | 0.772344 | 9.800865 | 3.070335 | 0.002853 | -1.7089 |
| UGDH | 0.594432 | 10.32376 | 3.068522 | 0.002869 | -1.71323 |
| TFG | 0.673451 | 9.847318 | 3.067818 | 0.002875 | -1.71491 |
| KRT222 | -0.54276 | 6.079385 | -3.06472 | 0.002902 | -1.72229 |
| VNN3 | -0.66052 | 5.895014 | -3.05831 | 0.002959 | -1.73757 |
| MYOT | -0.71744 | 6.407343 | -3.05655 | 0.002974 | -1.74177 |
| IL18RAP | -0.84996 | 6.847196 | -3.05495 | 0.002989 | -1.74558 |
| MYO5C | -0.63415 | 7.972927 | -3.0505 | 0.003029 | -1.75616 |
| TMEM97 | 0.579873 | 9.498762 | 3.046365 | 0.003067 | -1.76598 |
| TRDMT1 | -0.5295 | 7.747638 | -3.04616 | 0.003069 | -1.76646 |
| CLEC2D | -0.52435 | 8.437189 | -3.03651 | 0.003159 | -1.78934 |
| FADS2 | 1.032609 | 8.77928 | 3.029285 | 0.003228 | -1.80644 |
| FANCB | 0.76639 | 7.999003 | 3.027883 | 0.003241 | -1.80975 |
| MS4A3 | -0.88189 | 7.03632 | -3.02384 | 0.003281 | -1.81931 |
| GART | 0.579733 | 9.654313 | 3.020152 | 0.003317 | -1.828 |
| PRKCSH | 0.571743 | 9.769558 | 3.016502 | 0.003353 | -1.8366 |
| KIAA0040 | -0.52523 | 8.864988 | -3.0137 | 0.003381 | -1.84319 |
| REXO4 | 0.528833 | 8.373395 | 3.013571 | 0.003383 | -1.8435 |
| GSTM5 | -0.6691 | 6.681774 | -3.01223 | 0.003396 | -1.84666 |
| GAL3ST4 | 0.545783 | 8.212238 | 3.009544 | 0.003423 | -1.85298 |
| ISG15 | 0.976577 | 6.961219 | 3.009381 | 0.003425 | -1.85336 |
| ULBP2 | 0.733336 | 5.991631 | 3.007382 | 0.003446 | -1.85806 |
| ZBTB20 | -0.75682 | 8.860425 | -3.0032 | 0.003489 | -1.86789 |
| OR52B6 | -0.50252 | 5.783626 | -3.00235 | 0.003498 | -1.86989 |
| AGPAT4 | -0.69059 | 8.399877 | -3.00177 | 0.003504 | -1.87125 |
| SEC61A1 | 0.729677 | 11.81748 | 3.001623 | 0.003505 | -1.87159 |
| DSEL | 0.791949 | 10.67525 | 2.992184 | 0.003605 | -1.89371 |
| WFIKKN1 | -0.58353 | 5.419469 | -2.98846 | 0.003645 | -1.90242 |
| C19orf38 | -0.65353 | 5.810718 | -2.98749 | 0.003655 | -1.9047 |
| SLC22A3 | -0.73573 | 6.907687 | -2.98697 | 0.003661 | -1.9059 |
| MGAM | -0.58459 | 6.97195 | -2.98687 | 0.003662 | -1.90614 |
| EPB49 | -0.63223 | 7.063098 | -2.98445 | 0.003688 | -1.91179 |
| FCRL3 | -0.55395 | 6.19586 | -2.97621 | 0.003779 | -1.93102 |
| LONRF1 | -0.75755 | 8.25596 | -2.97179 | 0.003829 | -1.94133 |
| OSTC | 0.750584 | 9.896588 | 2.970315 | 0.003846 | -1.94476 |
| WIF1 | -0.66513 | 7.706503 | -2.97006 | 0.003849 | -1.94535 |
| LTF | -0.7853 | 8.022243 | -2.96221 | 0.003939 | -1.96361 |
| CEACAM8 | -0.80366 | 7.076098 | -2.95832 | 0.003984 | -1.97264 |
| BTNL9 | -0.92523 | 7.395826 | -2.9501 | 0.004082 | -1.99169 |
| DNAJC3 | 0.726963 | 9.712016 | 2.94968 | 0.004087 | -1.99265 |
| PRO0611 | -0.50003 | 6.256834 | -2.94895 | 0.004095 | -1.99435 |
| STRA13 | 0.732802 | 8.770831 | 2.948694 | 0.004098 | -1.99493 |
| SNF8 | 0.529316 | 8.63878 | 2.946212 | 0.004128 | -2.00068 |
| S100A8 | -0.86359 | 8.707448 | -2.94602 | 0.004131 | -2.00112 |
| CA1 | -0.98295 | 7.813833 | -2.94465 | 0.004147 | -2.00428 |
| CTTNBP2 | -0.56524 | 7.215994 | -2.94435 | 0.004151 | -2.00499 |
| GPR182 | -0.57193 | 5.982691 | -2.94188 | 0.004181 | -2.01069 |
| FBXO5 | 0.621017 | 9.114749 | 2.938134 | 0.004227 | -2.01934 |
| KLF8 | -0.53558 | 7.462388 | -2.93712 | 0.00424 | -2.02167 |
| TXNDC6 | -0.54957 | 6.809884 | -2.93681 | 0.004244 | -2.02239 |
| ATXN2L | 0.567535 | 8.472184 | 2.935596 | 0.004259 | -2.02519 |
| CD99L2 | 0.557431 | 10.29214 | 2.932399 | 0.004299 | -2.03256 |
| MRPL17 | 0.544061 | 9.386917 | 2.930706 | 0.00432 | -2.03646 |
| HIF1A | 0.680698 | 10.92869 | 2.929274 | 0.004338 | -2.03976 |
| ISG20L2 | 0.541241 | 8.426863 | 2.927087 | 0.004366 | -2.04479 |
| CD248 | 0.920849 | 9.556786 | 2.927078 | 0.004366 | -2.04481 |
| NCLN | 0.619018 | 9.293655 | 2.92429 | 0.004402 | -2.05122 |
| ABCC8 | -0.5661 | 6.30991 | -2.91713 | 0.004495 | -2.06767 |
| RRBP1 | 0.845159 | 11.73054 | 2.913167 | 0.004547 | -2.07675 |
| HMCN1 | 0.764294 | 9.606999 | 2.911541 | 0.004569 | -2.08048 |
| STXBP6 | 0.855169 | 8.759493 | 2.907069 | 0.004629 | -2.09072 |
| TIMP4 | -0.82048 | 7.314718 | -2.90549 | 0.00465 | -2.09433 |
| SAMD3 | -0.5698 | 7.111689 | -2.90502 | 0.004656 | -2.09541 |
| CD244 | -0.51379 | 6.534344 | -2.90478 | 0.00466 | -2.09596 |
| SCYL1 | 0.598154 | 9.158144 | 2.904183 | 0.004668 | -2.09732 |
| MEGF8 | 0.536156 | 8.096322 | 2.903905 | 0.004671 | -2.09795 |
| COL25A1 | -0.71732 | 5.885517 | -2.90342 | 0.004678 | -2.09907 |
| ABAT | -0.52379 | 7.626122 | -2.90322 | 0.004681 | -2.09951 |
| RAN | 0.894525 | 10.31774 | 2.90123 | 0.004708 | -2.10407 |
| DMD | -0.74691 | 8.190419 | -2.89772 | 0.004756 | -2.11209 |
| PLAUR | 0.889278 | 8.458064 | 2.889177 | 0.004875 | -2.13156 |
| HSPA5 | 0.86979 | 12.04067 | 2.887712 | 0.004896 | -2.13489 |
| RNPC3 | -0.70079 | 8.719948 | -2.88739 | 0.004901 | -2.13562 |
| SLC25A18 | -0.58786 | 5.712607 | -2.88676 | 0.00491 | -2.13706 |
| VPS37B | -0.58479 | 7.215357 | -2.88562 | 0.004926 | -2.13965 |
| SEMA3A | 0.745662 | 8.083567 | 2.883853 | 0.004951 | -2.14367 |
| ARRDC2 | -0.61388 | 7.753166 | -2.87284 | 0.005111 | -2.16867 |
| TAS2R13 | -0.58162 | 6.025902 | -2.87064 | 0.005144 | -2.17365 |
| N4BP2L1 | -0.54207 | 7.056699 | -2.87009 | 0.005152 | -2.17491 |
| SLC24A5 | -0.61001 | 5.937201 | -2.86787 | 0.005185 | -2.17992 |
| PTK7 | 0.751517 | 9.005683 | 2.867379 | 0.005193 | -2.18104 |
| THBS3 | 0.662504 | 9.930874 | 2.866497 | 0.005206 | -2.18303 |
| MAGI1 | -0.57204 | 8.340432 | -2.86183 | 0.005276 | -2.19359 |
| ABCF1 | 0.562301 | 11.01956 | 2.853413 | 0.005405 | -2.21258 |
| SCUBE3 | 0.647105 | 8.054257 | 2.852819 | 0.005415 | -2.21392 |
| FOXP1 | -0.51037 | 9.28186 | -2.84817 | 0.005487 | -2.22439 |
| PPT1 | 0.837036 | 10.54272 | 2.847276 | 0.005502 | -2.2264 |
| KLK3 | -0.56703 | 5.737729 | -2.84598 | 0.005522 | -2.22933 |
| KRT1 | -0.61226 | 6.531246 | -2.84504 | 0.005537 | -2.23143 |
| KLHL6 | -0.50031 | 7.748322 | -2.84311 | 0.005568 | -2.23578 |
| PRTN3 | -0.69702 | 6.544046 | -2.84136 | 0.005596 | -2.2397 |
| TGFB3 | 1.06526 | 9.60969 | 2.838151 | 0.005647 | -2.24691 |
| EFTUD2 | 0.633592 | 8.60652 | 2.831385 | 0.005758 | -2.26208 |
| TMEM222 | 0.595151 | 7.323394 | 2.829673 | 0.005786 | -2.26592 |
| GLIS3 | 0.777369 | 8.152763 | 2.82535 | 0.005858 | -2.27559 |
| FRAT2 | -0.97897 | 7.983494 | -2.8252 | 0.00586 | -2.27593 |
| CTHRC1 | 0.754212 | 10.44387 | 2.822138 | 0.005912 | -2.28277 |
| PAQR6 | -0.80292 | 7.309502 | -2.82157 | 0.005921 | -2.28405 |
| BRI3BP | 0.686466 | 7.933577 | 2.821484 | 0.005923 | -2.28423 |
| GFPT1 | 0.667961 | 9.601794 | 2.817899 | 0.005984 | -2.29224 |
| GNG10 | 0.901958 | 7.984496 | 2.815169 | 0.00603 | -2.29833 |
| RTKN | 0.665035 | 7.782234 | 2.809265 | 0.006133 | -2.31148 |
| SLC15A2 | -0.51406 | 6.951089 | -2.80921 | 0.006134 | -2.31161 |
| TRIM58 | -0.55849 | 7.787491 | -2.80436 | 0.006219 | -2.32238 |
| OLFM4 | -0.85989 | 7.273911 | -2.80157 | 0.006268 | -2.32859 |
| ROR2 | 0.896537 | 8.626089 | 2.800934 | 0.006279 | -2.33 |
| EGFL6 | 0.832093 | 9.076408 | 2.7968 | 0.006353 | -2.33918 |
| LYPD1 | 0.537527 | 6.78509 | 2.795054 | 0.006385 | -2.34305 |
| ERBB3 | -0.53266 | 6.621786 | -2.79338 | 0.006415 | -2.34675 |
| IPO13 | 0.628332 | 8.447268 | 2.789693 | 0.006483 | -2.35493 |
| EMR3 | -0.69142 | 6.243163 | -2.78673 | 0.006537 | -2.36149 |
| COL17A1 | -0.65865 | 7.170965 | -2.78627 | 0.006546 | -2.3625 |
| PODN | 0.651224 | 8.32134 | 2.786023 | 0.00655 | -2.36305 |
| CDK2AP2 | 0.927297 | 8.037282 | 2.783903 | 0.00659 | -2.36773 |
| GP1BA | -0.63807 | 6.939004 | -2.78091 | 0.006646 | -2.37434 |
| ANKAR | -0.68364 | 8.526162 | -2.78035 | 0.006656 | -2.37559 |
| MLLT10 | -0.61511 | 8.75488 | -2.78027 | 0.006658 | -2.37576 |
| SLFN14 | -0.69745 | 6.688299 | -2.7778 | 0.006704 | -2.38121 |
| HAL | -0.50367 | 5.94299 | -2.77723 | 0.006715 | -2.38247 |
| ODZ2 | 0.595608 | 7.801639 | 2.775363 | 0.006751 | -2.38658 |
| ABCG8 | -0.60433 | 5.73072 | -2.77526 | 0.006752 | -2.3868 |
| SLC2A4 | -0.51456 | 6.443648 | -2.77486 | 0.00676 | -2.38769 |
| KIAA0922 | -0.58331 | 8.666556 | -2.7739 | 0.006778 | -2.38981 |
| CNDP2 | 0.738737 | 10.19472 | 2.773058 | 0.006795 | -2.39166 |
| CEACAM6 | -0.85098 | 6.997006 | -2.76994 | 0.006854 | -2.39852 |
| TMEM45A | 0.53333 | 7.353471 | 2.769539 | 0.006862 | -2.39941 |
| KLHDC5 | 0.673754 | 9.973812 | 2.765279 | 0.006945 | -2.40877 |
| SELL | -0.77325 | 7.717478 | -2.76392 | 0.006972 | -2.41177 |
| AMPH | 0.581765 | 7.315442 | 2.760607 | 0.007037 | -2.41904 |
| LAIR2 | -0.66272 | 5.517175 | -2.76032 | 0.007042 | -2.41966 |
| HBM | -0.63185 | 5.651586 | -2.75781 | 0.007092 | -2.42516 |
| S100A10 | 0.695105 | 10.81537 | 2.75659 | 0.007117 | -2.42785 |
| CD226 | -0.62659 | 7.046602 | -2.75416 | 0.007165 | -2.43318 |
| PRKCQ | -0.68735 | 7.375929 | -2.75383 | 0.007172 | -2.4339 |
| FLJ45340 | -0.63052 | 8.645854 | -2.75256 | 0.007198 | -2.43668 |
| S100A12 | -0.82209 | 7.418651 | -2.74978 | 0.007254 | -2.44275 |
| TNFRSF4 | 0.691797 | 7.68204 | 2.744309 | 0.007366 | -2.45472 |
| PNO1 | 0.697246 | 9.070203 | 2.740753 | 0.00744 | -2.46248 |
| EPYC | 0.674399 | 6.698485 | 2.739762 | 0.00746 | -2.46464 |
| EGLN3 | 0.635841 | 7.75777 | 2.739143 | 0.007473 | -2.46599 |
| STAB2 | -0.74569 | 6.530683 | -2.7384 | 0.007489 | -2.46762 |
| HLF | -0.5837 | 7.341295 | -2.73719 | 0.007514 | -2.47025 |
| B4GALNT1 | 0.619345 | 7.337639 | 2.736295 | 0.007533 | -2.4722 |
| C19orf33 | -0.5812 | 5.165003 | -2.73511 | 0.007558 | -2.47477 |
| PLP1 | -0.59398 | 6.885708 | -2.73405 | 0.00758 | -2.47709 |
| OR51Q1 | -0.52056 | 5.570277 | -2.72784 | 0.007713 | -2.49059 |
| INPP4B | -0.57723 | 8.254151 | -2.72506 | 0.007773 | -2.49665 |
| NKG7 | -0.7552 | 7.038651 | -2.72391 | 0.007798 | -2.49914 |
| CTSA | 0.801911 | 9.486459 | 2.723589 | 0.007805 | -2.49983 |
| CALU | 0.744104 | 10.94731 | 2.71793 | 0.007929 | -2.51211 |
| HCFC1R1 | 0.655965 | 8.78283 | 2.714659 | 0.008001 | -2.51919 |
| SCNM1 | 0.526361 | 8.075918 | 2.70908 | 0.008126 | -2.53126 |
| GLT25D1 | 0.584033 | 9.470189 | 2.704139 | 0.008238 | -2.54193 |
| MRPS21 | 0.767854 | 9.413197 | 2.703497 | 0.008253 | -2.54331 |
| ZDHHC17 | -0.50886 | 8.205586 | -2.69764 | 0.008388 | -2.55594 |
| PDCD5 | 0.502406 | 7.81994 | 2.695747 | 0.008432 | -2.56001 |
| CDA | -0.89968 | 7.168931 | -2.69433 | 0.008465 | -2.56306 |
| LOC550643 | 0.638488 | 10.7315 | 2.694234 | 0.008467 | -2.56327 |
| TCN1 | -0.54875 | 5.971454 | -2.69291 | 0.008498 | -2.56611 |
| ADAMTS20 | -0.55838 | 5.879881 | -2.68952 | 0.008578 | -2.5734 |
| HRC | -0.55845 | 6.682215 | -2.68766 | 0.008622 | -2.5774 |
| PRSS23 | 0.724533 | 10.25392 | 2.683123 | 0.008731 | -2.58713 |
| COL5A2 | 0.804179 | 11.30601 | 2.679717 | 0.008813 | -2.59443 |
| PRDX4 | 0.673538 | 10.22364 | 2.677459 | 0.008868 | -2.59926 |
| LOXHD1 | -0.57415 | 7.088154 | -2.67562 | 0.008913 | -2.6032 |
| TUBB1 | -0.75842 | 7.386355 | -2.67008 | 0.00905 | -2.61504 |
| YIPF1 | 0.569402 | 8.451832 | 2.668588 | 0.009087 | -2.61823 |
| LYZ | -0.66516 | 9.781075 | -2.65989 | 0.009307 | -2.63676 |
| CAMP | -0.86539 | 6.867099 | -2.65937 | 0.00932 | -2.63786 |
| PER1 | -0.54715 | 7.170665 | -2.65746 | 0.009369 | -2.64194 |
| NONO | 0.551491 | 10.77413 | 2.653845 | 0.009462 | -2.64962 |
| NPY5R | -0.57307 | 6.205888 | -2.65248 | 0.009497 | -2.65252 |
| SNAI2 | 0.88917 | 11.02208 | 2.645405 | 0.009683 | -2.66753 |
| GNL2 | 0.820968 | 10.27649 | 2.643305 | 0.009739 | -2.67198 |
| KIAA1407 | -0.77305 | 7.950184 | -2.64215 | 0.009769 | -2.67443 |
| DAPK2 | -0.5084 | 7.54236 | -2.64193 | 0.009775 | -2.67488 |
| FAH | 0.593893 | 7.242856 | 2.641799 | 0.009779 | -2.67517 |
| MPO | -0.90822 | 8.605121 | -2.63103 | 0.01007 | -2.69792 |
| UCHL1 | 0.979604 | 9.546593 | 2.62974 | 0.010105 | -2.70064 |
| WNT5A | 0.770012 | 8.344594 | 2.628527 | 0.010139 | -2.7032 |
| LPAR5 | 0.638487 | 7.641449 | 2.625247 | 0.010229 | -2.71011 |
| KCTD4 | -0.53677 | 6.21072 | -2.62179 | 0.010326 | -2.7174 |
| FAM84B | -0.74449 | 7.179732 | -2.61879 | 0.01041 | -2.7237 |
| SDC3 | 0.635819 | 9.034973 | 2.618462 | 0.01042 | -2.72439 |
| GORASP2 | 0.596646 | 11.68738 | 2.617688 | 0.010442 | -2.72601 |
| GFI1 | -0.66187 | 7.085288 | -2.61746 | 0.010448 | -2.72649 |
| CASC4 | 0.640926 | 10.45551 | 2.616576 | 0.010473 | -2.72835 |
| ELOVL4 | 0.756769 | 9.414361 | 2.615113 | 0.010515 | -2.73142 |
| PPP2R5A | -0.67129 | 8.553123 | -2.61345 | 0.010562 | -2.73491 |
| ACSL6 | -0.80809 | 6.76807 | -2.61181 | 0.010609 | -2.73834 |
| ABCA6 | -0.68615 | 8.229067 | -2.6118 | 0.010609 | -2.73836 |
| HSPA2 | 0.760406 | 8.18174 | 2.609619 | 0.010672 | -2.74294 |
| LOC100134259 | 0.576408 | 5.593426 | 2.608457 | 0.010706 | -2.74537 |
| RDBP | 0.590312 | 10.42505 | 2.608049 | 0.010718 | -2.74623 |
| SP4 | -0.50527 | 8.296672 | -2.6072 | 0.010742 | -2.748 |
| TGFBI | 0.683257 | 11.62325 | 2.606187 | 0.010772 | -2.75013 |
| NCCRP1 | 0.619343 | 6.082743 | 2.603488 | 0.010851 | -2.75577 |
| CASQ2 | -0.52341 | 7.032911 | -2.60311 | 0.010862 | -2.75655 |
| ATP8B2 | 0.559333 | 8.117369 | 2.600776 | 0.01093 | -2.76144 |
| CDH4 | -0.59693 | 6.042371 | -2.6003 | 0.010944 | -2.76243 |
| CHSY1 | 0.51874 | 10.91882 | 2.599824 | 0.010959 | -2.76343 |
| CRISP3 | -0.97848 | 7.256674 | -2.59793 | 0.011015 | -2.76737 |
| PRKAB1 | -0.61207 | 7.556278 | -2.59639 | 0.011061 | -2.77059 |
| TMOD4 | -0.64512 | 6.09784 | -2.5952 | 0.011096 | -2.77308 |
| KIR3DL2 | -0.77244 | 6.052966 | -2.59518 | 0.011097 | -2.77313 |
| KPNA2 | 0.618763 | 9.309568 | 2.595122 | 0.011098 | -2.77324 |
| LPPR4 | 0.527884 | 7.536337 | 2.594272 | 0.011124 | -2.77502 |
| FGD1 | 0.923879 | 9.289311 | 2.592515 | 0.011177 | -2.77868 |
| ZFP2 | -0.60113 | 6.65918 | -2.5921 | 0.011189 | -2.77954 |
| C5orf56 | -0.50351 | 7.141956 | -2.59205 | 0.011191 | -2.77964 |
| TIMM9 | 0.628806 | 8.189352 | 2.59088 | 0.011226 | -2.78208 |
| HSP90B1 | 0.635489 | 11.75984 | 2.590496 | 0.011238 | -2.78288 |
| ALG2 | 0.577833 | 8.307265 | 2.587755 | 0.011321 | -2.78859 |
| DDX5 | -0.62704 | 10.43886 | -2.58472 | 0.011414 | -2.79489 |
| BTG3 | 0.601793 | 9.076649 | 2.581948 | 0.011499 | -2.80066 |
| ACBD3 | 0.553769 | 9.537478 | 2.581776 | 0.011504 | -2.80101 |
| GNA12 | 0.653081 | 8.474707 | 2.579917 | 0.011562 | -2.80487 |
| ATP2A3 | -0.57017 | 7.241063 | -2.57857 | 0.011604 | -2.80767 |
| FSCN1 | 0.510889 | 10.09245 | 2.578371 | 0.01161 | -2.80808 |
| PLD3 | 0.58735 | 10.63818 | 2.57789 | 0.011625 | -2.80908 |
| C1QTNF6 | 0.688745 | 9.438752 | 2.574772 | 0.011723 | -2.81554 |
| SLCO4C1 | -0.59535 | 7.006156 | -2.57357 | 0.01176 | -2.81803 |
| DEFA4 | -0.71638 | 7.281724 | -2.57007 | 0.011871 | -2.82528 |
| PRG3 | -0.57946 | 5.870023 | -2.56934 | 0.011895 | -2.8268 |
| BPI | -0.59927 | 6.744554 | -2.56765 | 0.011949 | -2.83029 |
| PPBP | -0.70229 | 8.014929 | -2.56734 | 0.011958 | -2.83092 |
| C11orf21 | -0.5835 | 6.084695 | -2.56694 | 0.011971 | -2.83176 |
| GYPB | -0.55539 | 5.776979 | -2.56563 | 0.012013 | -2.83446 |
| PPARG | -0.61261 | 8.642537 | -2.56547 | 0.012018 | -2.83479 |
| CIRBP | -0.5895 | 9.566989 | -2.56541 | 0.01202 | -2.83491 |
| TUBB3 | 0.92157 | 8.51691 | 2.561798 | 0.012137 | -2.84237 |
| GPC1 | 0.551751 | 7.563148 | 2.561299 | 0.012153 | -2.8434 |
| MED16 | 0.540805 | 10.03066 | 2.557444 | 0.012279 | -2.85134 |
| MKRN1 | -0.51019 | 10.68227 | -2.5554 | 0.012346 | -2.85555 |
| SLC39A1 | 0.516176 | 9.026225 | 2.552822 | 0.012431 | -2.86086 |
| KLHL35 | 0.695657 | 7.488596 | 2.550526 | 0.012508 | -2.86558 |
| MESDC2 | 0.573642 | 9.140696 | 2.550177 | 0.012519 | -2.86629 |
| ANXA5 | 0.597257 | 9.844158 | 2.54903 | 0.012558 | -2.86865 |
| COL3A1 | 0.732572 | 13.0497 | 2.548531 | 0.012574 | -2.86968 |
| PITX2 | 0.771758 | 8.467911 | 2.548083 | 0.012589 | -2.8706 |
| GOLGA8A | -0.74522 | 7.538994 | -2.54771 | 0.012602 | -2.87136 |
| BIN2 | -0.56351 | 7.622668 | -2.54756 | 0.012607 | -2.87167 |
| DKC1 | 0.530733 | 10.04968 | 2.546294 | 0.012649 | -2.87427 |
| NCF1C | -0.76596 | 5.890684 | -2.54497 | 0.012694 | -2.87698 |
| ACTN3 | -0.60559 | 5.783718 | -2.53822 | 0.012924 | -2.89083 |
| LDLR | 0.59029 | 9.067735 | 2.53787 | 0.012936 | -2.89154 |
| C1orf204 | 0.511801 | 7.238138 | 2.537542 | 0.012947 | -2.89221 |
| WBP11 | 0.563431 | 8.712409 | 2.536011 | 0.013 | -2.89534 |
| SFXN3 | 0.549912 | 8.952623 | 2.534522 | 0.013051 | -2.89839 |
| CDKN2A | 1.040846 | 7.956217 | 2.532347 | 0.013127 | -2.90283 |
| GRAMD1A | 0.518493 | 8.581197 | 2.532147 | 0.013134 | -2.90324 |
| NR2C2AP | 0.589585 | 7.955637 | 2.527348 | 0.013302 | -2.91304 |
| DNASE1L3 | -0.63283 | 7.607364 | -2.52574 | 0.013359 | -2.91632 |
| PNMAL2 | -0.69708 | 6.51533 | -2.52557 | 0.013365 | -2.91666 |
| PDPN | 0.930684 | 9.73949 | 2.522056 | 0.013489 | -2.92382 |
| DNASE2 | 0.60934 | 8.032908 | 2.521376 | 0.013514 | -2.9252 |
| SFRS18 | -0.60884 | 9.940123 | -2.52112 | 0.013523 | -2.92572 |
| HSPA13 | 0.510186 | 9.836011 | 2.521039 | 0.013526 | -2.92589 |
| CITED2 | -0.73495 | 8.628244 | -2.51805 | 0.013633 | -2.93198 |
| SH3GL1 | 0.574374 | 9.85203 | 2.517982 | 0.013635 | -2.93211 |
| CARS | 0.549724 | 9.977627 | 2.517312 | 0.01366 | -2.93347 |
| LUZP1 | 0.625945 | 10.45347 | 2.514873 | 0.013748 | -2.93843 |
| HHLA3 | 0.784288 | 7.30068 | 2.514551 | 0.01376 | -2.93908 |
| CD200 | -0.50819 | 8.263457 | -2.51415 | 0.013774 | -2.9399 |
| EBF1 | -0.72467 | 9.129554 | -2.50989 | 0.01393 | -2.94854 |
| IMP4 | 0.54105 | 9.489153 | 2.509131 | 0.013958 | -2.95008 |
| SLC35A2 | 0.556296 | 9.389584 | 2.507701 | 0.01401 | -2.95298 |
| GPNMB | 0.514187 | 9.968645 | 2.506284 | 0.014063 | -2.95585 |
| TNFRSF10C | -0.52859 | 6.442637 | -2.50597 | 0.014074 | -2.95648 |
| PSMB5 | 0.721689 | 9.321543 | 2.503977 | 0.014148 | -2.96052 |
| RNASE3 | -0.66145 | 6.76151 | -2.5031 | 0.014181 | -2.96229 |
| COL2A1 | 1.307413 | 8.711294 | 2.501877 | 0.014227 | -2.96477 |
| VRK2 | 0.524109 | 9.107131 | 2.500951 | 0.014261 | -2.96664 |
| ADHFE1 | -0.66356 | 6.870523 | -2.50006 | 0.014295 | -2.96844 |
| NDUFA4L2 | 0.909819 | 7.388284 | 2.497853 | 0.014378 | -2.9729 |
| AGPAT6 | 0.536447 | 8.673018 | 2.494194 | 0.014517 | -2.98028 |
| DDRGK1 | 0.612364 | 8.398204 | 2.491834 | 0.014607 | -2.98504 |
| DGAT2 | -0.58081 | 6.847277 | -2.49167 | 0.014613 | -2.98537 |
| C5orf40 | -0.64976 | 6.714014 | -2.49155 | 0.014618 | -2.98562 |
| TBX21 | -0.51493 | 6.468204 | -2.49154 | 0.014618 | -2.98564 |
| ABCA8 | -0.83892 | 9.359915 | -2.49057 | 0.014655 | -2.98758 |
| ZMAT1 | -0.65834 | 7.975448 | -2.48989 | 0.014682 | -2.98895 |
| ZNF48 | -0.52565 | 6.065333 | -2.48872 | 0.014727 | -2.99131 |
| GYPA | -0.62796 | 7.278545 | -2.48611 | 0.014828 | -2.99657 |
| DPYSL4 | 0.774423 | 7.228826 | 2.480674 | 0.01504 | -3.00749 |
| SSU72 | 0.724869 | 9.983726 | 2.479039 | 0.015105 | -3.01077 |
| APH1A | 0.512261 | 7.588006 | 2.47698 | 0.015186 | -3.0149 |
| ADD2 | -0.64378 | 7.42396 | -2.47676 | 0.015195 | -3.01534 |
| LOC399959 | -0.63268 | 8.648675 | -2.47438 | 0.01529 | -3.02011 |
| WIPI1 | 0.654417 | 8.985983 | 2.473973 | 0.015306 | -3.02093 |
| MEPE | -1.16012 | 10.02854 | -2.47241 | 0.015369 | -3.02406 |
| MS4A1 | -0.53064 | 6.929146 | -2.47214 | 0.015379 | -3.0246 |
| C9orf102 | -0.57353 | 8.342881 | -2.47157 | 0.015402 | -3.02574 |
| GRHL2 | -0.59665 | 6.064557 | -2.47047 | 0.015447 | -3.02795 |
| FAM111B | 0.614276 | 8.168732 | 2.468468 | 0.015527 | -3.03195 |
| SC4MOL | 0.640638 | 9.871999 | 2.465529 | 0.015647 | -3.03782 |
| PHF20L1 | 0.594998 | 11.03141 | 2.464002 | 0.015709 | -3.04087 |
| GGT8P | -0.52962 | 5.704729 | -2.45918 | 0.015907 | -3.05049 |
| CNOT3 | 0.637213 | 8.180214 | 2.458156 | 0.01595 | -3.05253 |
| PRAF2 | 0.561374 | 8.480455 | 2.457687 | 0.015969 | -3.05346 |
| DNAJC5G | -0.72318 | 5.924849 | -2.4565 | 0.016018 | -3.05582 |
| HAPLN1 | 0.81671 | 8.929197 | 2.453629 | 0.016138 | -3.06154 |
| STC2 | 0.723455 | 8.739295 | 2.453287 | 0.016153 | -3.06222 |
| RCN3 | 0.740177 | 9.025341 | 2.448612 | 0.01635 | -3.07151 |
| HIST1H2BK | 0.545844 | 8.857414 | 2.44853 | 0.016353 | -3.07167 |
| GJB2 | 0.671727 | 7.097999 | 2.445896 | 0.016465 | -3.0769 |
| GALNT4 | -0.50098 | 7.846612 | -2.44582 | 0.016468 | -3.07704 |
| ZBTB2 | 0.543124 | 8.670786 | 2.44231 | 0.016619 | -3.08401 |
| TNFAIP8L3 | 0.509389 | 7.542716 | 2.440958 | 0.016677 | -3.08669 |
| SLC25A32 | 0.654697 | 9.150647 | 2.439686 | 0.016732 | -3.08921 |
| GDPD5 | -0.60101 | 7.27864 | -2.43955 | 0.016738 | -3.08947 |
| TRIP13 | 0.755501 | 7.671236 | 2.437452 | 0.016829 | -3.09363 |
| COL4A2 | 0.610144 | 9.651505 | 2.433321 | 0.01701 | -3.10179 |
| ZMYM1 | 0.565031 | 8.854347 | 2.432294 | 0.017055 | -3.10382 |
| UTP11L | 0.503418 | 9.327574 | 2.432061 | 0.017065 | -3.10428 |
| MPHOSPH10 | 0.587277 | 10.54708 | 2.430096 | 0.017152 | -3.10816 |
| PKIA | -0.65713 | 7.129497 | -2.42885 | 0.017207 | -3.11061 |
| CCNDBP1 | -0.68404 | 8.252569 | -2.4288 | 0.017209 | -3.11072 |
| ARHGAP22 | 0.544328 | 7.234629 | 2.422514 | 0.01749 | -3.1231 |
| SH3BGRL2 | -0.55486 | 8.583602 | -2.42174 | 0.017525 | -3.12461 |
| NDUFAB1 | 0.601721 | 10.59109 | 2.421411 | 0.01754 | -3.12527 |
| FCRLA | -0.50293 | 6.217086 | -2.41667 | 0.017755 | -3.13459 |
| RAB5A | 0.582331 | 9.310875 | 2.411669 | 0.017984 | -3.14439 |
| EPB42 | -0.65886 | 6.785919 | -2.41079 | 0.018025 | -3.14611 |
| C12orf11 | 0.54563 | 8.439143 | 2.409516 | 0.018084 | -3.14861 |
| P2RX1 | -0.66106 | 6.729623 | -2.4092 | 0.018099 | -3.14924 |
| BASP1 | 0.716959 | 10.60887 | 2.405667 | 0.018263 | -3.15615 |
| MSX1 | 0.654061 | 8.89226 | 2.404052 | 0.018339 | -3.1593 |
| PLEKHF1 | 0.793096 | 7.031196 | 2.40247 | 0.018413 | -3.1624 |
| PYGM | -0.81004 | 6.923078 | -2.40184 | 0.018443 | -3.16362 |
| CMTM2 | -0.64744 | 5.857134 | -2.39975 | 0.018542 | -3.1677 |
| UFC1 | 0.509741 | 9.13837 | 2.399448 | 0.018556 | -3.1683 |
| METTL1 | 0.617628 | 9.24359 | 2.399275 | 0.018564 | -3.16863 |
| FABP4 | -0.65497 | 8.895264 | -2.39854 | 0.018599 | -3.17008 |
| FCER1G | 0.773935 | 9.651153 | 2.395287 | 0.018755 | -3.17641 |
| SLC39A7 | 0.569552 | 8.517686 | 2.395235 | 0.018757 | -3.17651 |
| HRAS | 0.739562 | 8.401302 | 2.394462 | 0.018794 | -3.17802 |
| CTSB | 0.60312 | 12.76852 | 2.394203 | 0.018807 | -3.17852 |
| CHM | 0.510191 | 8.40756 | 2.392776 | 0.018875 | -3.1813 |
| NUP133 | -0.5653 | 8.309883 | -2.3919 | 0.018917 | -3.183 |
| HAVCR2 | 0.755697 | 9.273728 | 2.389394 | 0.019039 | -3.18788 |
| C17orf68 | -0.55017 | 8.079213 | -2.38595 | 0.019207 | -3.19458 |
| PSMC4 | 0.64125 | 8.257924 | 2.385074 | 0.019249 | -3.19627 |
| SHE | -0.52716 | 8.171839 | -2.38367 | 0.019318 | -3.19899 |
| TMEM107 | -0.71224 | 8.146147 | -2.38005 | 0.019497 | -3.20602 |
| GATA1 | -0.51846 | 5.773993 | -2.37681 | 0.019658 | -3.21229 |
| PPIC | 0.780445 | 10.59754 | 2.375026 | 0.019748 | -3.21575 |
| SLC6A16 | -0.58079 | 7.418039 | -2.3742 | 0.019789 | -3.21734 |
| MFI2 | 0.756253 | 7.516688 | 2.370326 | 0.019985 | -3.22483 |
| MID1 | 0.650669 | 9.730139 | 2.369619 | 0.020021 | -3.2262 |
| CADM1 | 0.77374 | 11.04422 | 2.36878 | 0.020063 | -3.22782 |
| PGAM4 | 0.583209 | 8.040152 | 2.368586 | 0.020073 | -3.22819 |
| COMP | 1.094772 | 7.721528 | 2.368217 | 0.020092 | -3.2289 |
| GRP | 1.028248 | 8.046513 | 2.366575 | 0.020176 | -3.23207 |
| CYP3A7 | -0.55341 | 5.531047 | -2.36423 | 0.020296 | -3.2366 |
| HMGCS1 | 0.587433 | 9.823702 | 2.363976 | 0.020309 | -3.23708 |
| CPEB3 | -0.5584 | 8.69469 | -2.36288 | 0.020365 | -3.23919 |
| TCF7L2 | -0.51379 | 8.737726 | -2.36153 | 0.020435 | -3.24179 |
| KLK2 | -0.50713 | 6.217023 | -2.36139 | 0.020442 | -3.24206 |
| FAM110B | 0.852286 | 9.202084 | 2.359674 | 0.020531 | -3.24536 |
| NEIL1 | -0.53297 | 6.659257 | -2.35494 | 0.020778 | -3.25447 |
| CLC | -0.78534 | 7.228032 | -2.3535 | 0.020854 | -3.25722 |
| SLC7A8 | 0.632315 | 8.876291 | 2.352021 | 0.020932 | -3.26006 |
| SS18 | 0.500067 | 10.13447 | 2.351632 | 0.020952 | -3.26081 |
| YIPF5 | 0.673997 | 9.976244 | 2.349916 | 0.021043 | -3.2641 |
| DDX39 | 0.809277 | 9.188413 | 2.349783 | 0.02105 | -3.26435 |
| PTTG1IP | 0.658183 | 10.86622 | 2.341806 | 0.021477 | -3.27962 |
| LENG8 | -0.63822 | 9.003177 | -2.34174 | 0.021481 | -3.27974 |
| LCMT1 | 0.551318 | 9.45773 | 2.339611 | 0.021596 | -3.28381 |
| FPR3 | 0.656996 | 8.982615 | 2.339182 | 0.02162 | -3.28463 |
| KCNB1 | -0.53825 | 7.247732 | -2.33908 | 0.021625 | -3.28483 |
| DYNLT3 | 0.573342 | 9.415718 | 2.338299 | 0.021668 | -3.28632 |
| FARP1 | 0.560272 | 8.962545 | 2.335036 | 0.021846 | -3.29254 |
| SNX22 | 0.575367 | 9.552192 | 2.333043 | 0.021956 | -3.29634 |
| RHAG | -0.51888 | 6.503703 | -2.32869 | 0.022196 | -3.30462 |
| ABHD5 | -0.58096 | 8.254832 | -2.32779 | 0.022247 | -3.30634 |
| HSF5 | -0.5229 | 6.170549 | -2.32534 | 0.022383 | -3.31099 |
| FCGR3B | -0.60705 | 7.301091 | -2.32328 | 0.022499 | -3.31491 |
| OR2B11 | -0.59764 | 5.401516 | -2.32322 | 0.022503 | -3.31502 |
| PEPD | 0.604699 | 8.23183 | 2.320803 | 0.022639 | -3.3196 |
| COL6A2 | 0.544593 | 8.774849 | 2.319067 | 0.022738 | -3.3229 |
| VARS | 0.685064 | 8.279387 | 2.317833 | 0.022808 | -3.32523 |
| CBFB | 0.579544 | 10.07579 | 2.313581 | 0.023051 | -3.33328 |
| PPP1R8 | 0.576346 | 8.928937 | 2.313556 | 0.023053 | -3.33333 |
| C1QTNF7 | -0.51672 | 7.581111 | -2.31199 | 0.023143 | -3.33628 |
| PTK2B | -0.50691 | 8.377678 | -2.30958 | 0.023282 | -3.34085 |
| TRIB2 | 0.507674 | 9.190885 | 2.307454 | 0.023406 | -3.34485 |
| FKBP11 | 0.590033 | 9.111585 | 2.307181 | 0.023422 | -3.34537 |
| KLF1 | -0.58615 | 6.596394 | -2.30542 | 0.023525 | -3.34869 |
| COL5A1 | 0.508328 | 11.35125 | 2.30481 | 0.023561 | -3.34984 |
| SCOC | 0.535547 | 8.431371 | 2.302167 | 0.023716 | -3.35482 |
| ZDHHC20 | 0.535406 | 8.7217 | 2.301831 | 0.023736 | -3.35545 |
| C5orf32 | -0.56453 | 7.82855 | -2.30127 | 0.023769 | -3.35651 |
| PTCD1 | 0.547612 | 9.741806 | 2.298725 | 0.02392 | -3.3613 |
| PRKAR2B | -0.59771 | 7.939877 | -2.29712 | 0.024015 | -3.36431 |
| HLA-DMA | 0.593935 | 9.840742 | 2.294364 | 0.02418 | -3.36949 |
| SERPIND1 | -0.64378 | 6.03313 | -2.28903 | 0.024502 | -3.3795 |
| ZFP36L2 | -0.53428 | 9.520503 | -2.28787 | 0.024572 | -3.38167 |
| SLAMF8 | 0.746083 | 8.118183 | 2.287423 | 0.0246 | -3.38251 |
| PPPDE1 | 0.607558 | 9.060488 | 2.283654 | 0.02483 | -3.38956 |
| HEXA | 0.511431 | 8.663848 | 2.283274 | 0.024854 | -3.39027 |
| CXCL16 | 0.757155 | 10.50407 | 2.282302 | 0.024913 | -3.39208 |
| PDDC1 | -0.62526 | 7.637238 | -2.28091 | 0.024999 | -3.39468 |
| HIST1H2BC | 0.629085 | 9.76369 | 2.280831 | 0.025004 | -3.39483 |
| PQLC2 | 0.729201 | 7.841004 | 2.276983 | 0.025243 | -3.40202 |
| RASSF5 | -0.5648 | 9.179566 | -2.2763 | 0.025285 | -3.40329 |
| CST7 | -0.50688 | 7.409713 | -2.27559 | 0.02533 | -3.40462 |
| CHKB | -0.52934 | 8.508417 | -2.27492 | 0.025372 | -3.40587 |
| GFRA1 | -0.52278 | 8.169655 | -2.2738 | 0.025442 | -3.40794 |
| BCL2L15 | -0.50018 | 6.676494 | -2.27377 | 0.025444 | -3.408 |
| ASAP3 | 0.748906 | 9.50744 | 2.268471 | 0.025778 | -3.41787 |
| MSTO2P | 0.68541 | 6.29741 | 2.26637 | 0.025912 | -3.42177 |
| NPY1R | -0.66198 | 6.850016 | -2.26391 | 0.026069 | -3.42634 |
| ACCN2 | 0.623857 | 7.699847 | 2.263142 | 0.026118 | -3.42776 |
| FAM180A | 0.582248 | 6.650868 | 2.263055 | 0.026124 | -3.42792 |
| DAP | 0.612111 | 9.32799 | 2.260796 | 0.026269 | -3.43211 |
| GAL3ST3 | -0.60889 | 6.106236 | -2.25948 | 0.026354 | -3.43455 |
| PTGIS | -0.50273 | 8.213943 | -2.25825 | 0.026434 | -3.43682 |
| CREB3L1 | 0.687219 | 10.28487 | 2.257907 | 0.026457 | -3.43746 |
| GATS | -0.54158 | 7.857607 | -2.25781 | 0.026463 | -3.43765 |
| SLC39A9 | 0.513384 | 9.349752 | 2.256413 | 0.026554 | -3.44023 |
| INADL | 0.622402 | 8.73795 | 2.251037 | 0.026906 | -3.45017 |
| DNTT | -0.70395 | 5.880073 | -2.25061 | 0.026934 | -3.45096 |
| MLLT11 | 0.555342 | 8.042729 | 2.248805 | 0.027053 | -3.45429 |
| SNAP25 | 0.825599 | 8.116515 | 2.245241 | 0.02729 | -3.46086 |
| SORCS1 | -0.68394 | 7.489141 | -2.2414 | 0.027548 | -3.46793 |
| PJA1 | 0.520655 | 7.803487 | 2.241347 | 0.027551 | -3.46803 |
| PTGER3 | -0.5205 | 7.635618 | -2.24133 | 0.027552 | -3.46805 |
| AKAP6 | -0.52794 | 8.389521 | -2.24127 | 0.027556 | -3.46816 |
| FCGR2A | 0.58758 | 8.793119 | 2.239424 | 0.027681 | -3.47156 |
| TMEM51 | 0.598455 | 7.878196 | 2.237048 | 0.027842 | -3.47593 |
| SPON2 | 0.587067 | 6.761781 | 2.234829 | 0.027993 | -3.48 |
| C6 | -0.50158 | 6.223202 | -2.23264 | 0.028143 | -3.48401 |
| SAC3D1 | 0.546207 | 7.450832 | 2.231391 | 0.028229 | -3.4863 |
| TIMELESS | 0.627002 | 9.020265 | 2.231008 | 0.028255 | -3.48701 |
| RTN1 | 0.61281 | 7.959022 | 2.226322 | 0.028579 | -3.49558 |
| APOD | -0.54161 | 8.159478 | -2.22561 | 0.028629 | -3.49689 |
| PSMB2 | 0.709208 | 9.414707 | 2.225341 | 0.028647 | -3.49738 |
| CIRH1A | 0.526524 | 10.47489 | 2.225221 | 0.028655 | -3.4976 |
| SNAPC5 | 0.502401 | 8.287402 | 2.223454 | 0.028779 | -3.50083 |
| GPN2 | 0.588455 | 9.45525 | 2.221929 | 0.028886 | -3.50361 |
| CRTAP | 0.605377 | 9.993281 | 2.220821 | 0.028963 | -3.50563 |
| SKP2 | 0.584471 | 9.16579 | 2.218833 | 0.029103 | -3.50926 |
| ATP2B2 | -0.50781 | 6.669769 | -2.21883 | 0.029104 | -3.50926 |
| C5orf4 | -0.50077 | 8.206315 | -2.21818 | 0.02915 | -3.51046 |
| MECR | 0.608582 | 7.084105 | 2.216766 | 0.02925 | -3.51303 |
| TMEM5 | 0.539619 | 8.046526 | 2.213361 | 0.029492 | -3.51922 |
| MGC12982 | 0.515732 | 6.757598 | 2.212983 | 0.029519 | -3.51991 |
| IKZF3 | -0.58473 | 7.634258 | -2.21147 | 0.029627 | -3.52266 |
| TMEM59 | 0.59918 | 11.10946 | 2.210719 | 0.029681 | -3.52403 |
| DAAM1 | -0.51075 | 8.797919 | -2.21041 | 0.029703 | -3.52459 |
| ZWINT | 0.572893 | 8.092414 | 2.208638 | 0.029831 | -3.52781 |
| FCN1 | -0.65411 | 7.587306 | -2.20715 | 0.029939 | -3.53051 |
| PPIF | 0.69671 | 8.837162 | 2.206977 | 0.029951 | -3.53083 |
| LMNB2 | 0.589455 | 8.013506 | 2.20594 | 0.030026 | -3.53271 |
| JMJD1C | -0.54965 | 9.938537 | -2.20418 | 0.030154 | -3.5359 |
| RNF216L | 0.602156 | 6.973601 | 2.20402 | 0.030166 | -3.53619 |
| MALAT1 | -0.71825 | 12.65852 | -2.20129 | 0.030365 | -3.54113 |
| SPTB | -0.80144 | 7.542045 | -2.19884 | 0.030545 | -3.54556 |
| DYDC2 | -0.61573 | 5.863734 | -2.19805 | 0.030603 | -3.54699 |
| SLC4A1 | -0.69293 | 7.896986 | -2.19721 | 0.030666 | -3.54852 |
| MYH11 | -0.84546 | 9.419915 | -2.19719 | 0.030667 | -3.54855 |
| RRAS | 0.594111 | 8.411955 | 2.196751 | 0.0307 | -3.54935 |
| RAB5C | 0.559122 | 8.922309 | 2.193628 | 0.030931 | -3.55499 |
| GJA1 | 0.673804 | 11.15633 | 2.192661 | 0.031004 | -3.55673 |
| SCARNA5 | -0.67413 | 7.008448 | -2.19225 | 0.031034 | -3.55748 |
| AHSP | -0.8049 | 7.582057 | -2.18801 | 0.031352 | -3.56512 |
| THY1 | 0.613157 | 9.151158 | 2.186103 | 0.031496 | -3.56855 |
| SMC1A | 0.607675 | 10.59286 | 2.183698 | 0.031679 | -3.57288 |
| DDB1 | 0.59697 | 10.01927 | 2.180005 | 0.031961 | -3.57951 |
| CA4 | -0.58365 | 5.761497 | -2.17984 | 0.031974 | -3.57981 |
| GINS2 | 0.728626 | 8.701022 | 2.17659 | 0.032223 | -3.58564 |
| ITGB3 | -0.5176 | 9.370231 | -2.17643 | 0.032236 | -3.58592 |
| C18orf19 | 0.604682 | 8.31525 | 2.175001 | 0.032346 | -3.58849 |
| THOC5 | 0.680515 | 10.66003 | 2.173854 | 0.032435 | -3.59054 |
| PDGFRB | 0.677816 | 10.62748 | 2.172634 | 0.03253 | -3.59272 |
| POLR1E | 0.502538 | 9.330456 | 2.171879 | 0.032589 | -3.59408 |
| IL1RAPL1 | -0.59914 | 6.095196 | -2.17086 | 0.032668 | -3.59591 |
| FKBP9 | 0.553005 | 9.125334 | 2.169569 | 0.032769 | -3.59821 |
| SCAMP3 | 0.616644 | 10.05308 | 2.16847 | 0.032855 | -3.60017 |
| SPHK1 | 0.536185 | 7.14391 | 2.166738 | 0.032991 | -3.60327 |
| ZNF362 | 0.625652 | 8.678078 | 2.16613 | 0.033039 | -3.60435 |
| CD177 | -0.55346 | 6.374884 | -2.1654 | 0.033097 | -3.60566 |
| INHBA | 0.622482 | 8.878832 | 2.164207 | 0.033191 | -3.60778 |
| NCKAP1 | 0.514584 | 9.530788 | 2.162778 | 0.033305 | -3.61033 |
| SLC35B4 | 0.51853 | 9.414355 | 2.16091 | 0.033453 | -3.61366 |
| G6PC | -0.51252 | 5.863714 | -2.16053 | 0.033483 | -3.61433 |
| GABRB1 | -0.53754 | 5.903431 | -2.15862 | 0.033636 | -3.61774 |
| VSNL1 | 0.713807 | 7.18707 | 2.15853 | 0.033644 | -3.6179 |
| VIT | -0.50601 | 7.668446 | -2.15802 | 0.033685 | -3.61881 |
| PSG1 | -0.5416 | 5.406195 | -2.15717 | 0.033753 | -3.62032 |
| ERF | 0.534062 | 7.426744 | 2.153171 | 0.034075 | -3.62742 |
| SNUPN | 0.53743 | 8.489186 | 2.15124 | 0.034232 | -3.63085 |
| TEX14 | -0.55532 | 6.522037 | -2.14836 | 0.034467 | -3.63596 |
| GFI1B | -0.61823 | 6.403107 | -2.14552 | 0.0347 | -3.64098 |
| PON3 | -0.85397 | 7.678214 | -2.14529 | 0.034719 | -3.6414 |
| RHBDF2 | 0.573011 | 8.944787 | 2.143587 | 0.03486 | -3.64441 |
| ADH1A | -0.51766 | 5.281582 | -2.14257 | 0.034944 | -3.64621 |
| ECT2 | 0.526867 | 9.486566 | 2.142397 | 0.034958 | -3.64651 |
| MAX | 0.506955 | 9.966291 | 2.13889 | 0.03525 | -3.65271 |
| LOC100190986 | -0.58129 | 7.835488 | -2.1384 | 0.035291 | -3.65358 |
| JAG2 | 0.684757 | 8.079325 | 2.135159 | 0.035562 | -3.65929 |
| KIRREL | 0.556715 | 8.420243 | 2.133058 | 0.035739 | -3.66299 |
| ANXA3 | -0.60967 | 6.835483 | -2.13245 | 0.035791 | -3.66407 |
| ABCA1 | 0.677489 | 11.44147 | 2.128405 | 0.036134 | -3.67118 |
| NCRNA00219 | -0.591 | 7.55479 | -2.12801 | 0.036168 | -3.67187 |
| TCOF1 | 0.544817 | 8.92194 | 2.124491 | 0.036469 | -3.67805 |
| MAN1B1 | 0.516485 | 7.859557 | 2.119982 | 0.036858 | -3.68596 |
| FAM86C | 0.585804 | 6.656832 | 2.118702 | 0.03697 | -3.6882 |
| PPP4R4 | 0.512953 | 6.598938 | 2.117979 | 0.037033 | -3.68946 |
| CDCP1 | 0.540384 | 7.833309 | 2.117064 | 0.037112 | -3.69106 |
| AQP7 | -0.75438 | 5.938968 | -2.11585 | 0.037218 | -3.69319 |
| FLAD1 | 0.605785 | 8.759065 | 2.114065 | 0.037375 | -3.69631 |
| SCHIP1 | -0.56124 | 7.735352 | -2.11296 | 0.037472 | -3.69824 |
| SLC29A4 | 0.659394 | 7.870236 | 2.110764 | 0.037665 | -3.70207 |
| RAGE | 0.62139 | 7.916943 | 2.10226 | 0.038424 | -3.71688 |
| HNRNPAB | 0.619954 | 10.84 | 2.101987 | 0.038448 | -3.71735 |
| ACAN | 0.809954 | 9.082426 | 2.098608 | 0.038753 | -3.72322 |
| TST | -0.50171 | 7.263507 | -2.09817 | 0.038792 | -3.72398 |
| C17orf58 | 0.593361 | 7.946306 | 2.097278 | 0.038874 | -3.72553 |
| CSAD | -0.53863 | 7.680186 | -2.09662 | 0.038934 | -3.72667 |
| MGAT4B | 0.543896 | 7.181647 | 2.092871 | 0.039276 | -3.73317 |
| NOP14 | 0.557976 | 10.17948 | 2.091411 | 0.03941 | -3.7357 |
| UBA2 | 0.503063 | 9.089919 | 2.086841 | 0.039832 | -3.7436 |
| PEA15 | 0.509645 | 12.35439 | 2.085816 | 0.039927 | -3.74537 |
| TMEM170B | -0.52122 | 8.127161 | -2.0857 | 0.039938 | -3.74557 |
| C14orf153 | 0.631394 | 8.726912 | 2.08383 | 0.040112 | -3.74879 |
| MYOM3 | -0.52891 | 6.554507 | -2.08361 | 0.040133 | -3.74918 |
| DNASE2B | -0.52561 | 6.210097 | -2.0831 | 0.04018 | -3.75005 |
| RPS19BP1 | 0.611143 | 7.164506 | 2.080362 | 0.040437 | -3.75477 |
| FBXL20 | -0.70274 | 9.996013 | -2.07956 | 0.040513 | -3.75616 |
| MAGEC2 | 0.566309 | 5.952451 | 2.076872 | 0.040766 | -3.76078 |
| PRDM14 | -0.62285 | 6.058768 | -2.07346 | 0.04109 | -3.76664 |
| CPEB1 | -0.54286 | 6.559981 | -2.07154 | 0.041273 | -3.76994 |
| TNFRSF25 | -0.60435 | 6.380422 | -2.06996 | 0.041424 | -3.77264 |
| CD74 | 0.579457 | 12.29766 | 2.069808 | 0.041439 | -3.77291 |
| APOB | -0.55853 | 7.218205 | -2.06819 | 0.041595 | -3.77568 |
| LPAR4 | 0.51243 | 8.045372 | 2.066841 | 0.041725 | -3.77799 |
| NSDHL | 0.644754 | 9.497021 | 2.066562 | 0.041752 | -3.77847 |
| CRABP2 | 0.684103 | 8.604878 | 2.066075 | 0.041799 | -3.7793 |
| ADAR | 0.617868 | 11.64441 | 2.064506 | 0.041951 | -3.78199 |
| RPL23AP7 | 0.738841 | 7.171795 | 2.061963 | 0.042198 | -3.78634 |
| DKK3 | 0.694093 | 10.40994 | 2.058052 | 0.042581 | -3.79301 |
| C17orf53 | 0.65012 | 8.512842 | 2.057705 | 0.042615 | -3.7936 |
| WBP5 | 0.598119 | 9.990471 | 2.055876 | 0.042795 | -3.79672 |
| C6orf125 | 0.547198 | 7.542642 | 2.054983 | 0.042883 | -3.79824 |
| CASQ1 | -0.53078 | 6.141099 | -2.05446 | 0.042935 | -3.79914 |
| SDHAP2 | 0.694254 | 7.316887 | 2.051514 | 0.043227 | -3.80415 |
| TAF13 | 0.609375 | 10.29408 | 2.045696 | 0.04381 | -3.81403 |
| ZFP64 | 0.519854 | 7.418886 | 2.045128 | 0.043867 | -3.81499 |
| ADIPOQ | -0.83319 | 8.634061 | -2.04424 | 0.043957 | -3.81651 |
| COL4A1 | 0.616002 | 12.00945 | 2.044138 | 0.043967 | -3.81667 |
| PRKCB | -0.51784 | 8.814011 | -2.04224 | 0.044158 | -3.81988 |
| PPFIBP1 | 0.596797 | 10.18148 | 2.041041 | 0.04428 | -3.82192 |
| SAE1 | 0.544553 | 10.9505 | 2.04095 | 0.04429 | -3.82208 |
| FAM46C | -0.60885 | 9.162561 | -2.03974 | 0.044413 | -3.82412 |
| MS4A4A | 0.58134 | 10.02646 | 2.038836 | 0.044505 | -3.82565 |
| IFI27L1 | 0.570479 | 8.236221 | 2.037034 | 0.044689 | -3.8287 |
| GLS2 | -0.57278 | 6.590922 | -2.03319 | 0.045084 | -3.83519 |
| SGK196 | 0.562684 | 7.721832 | 2.028146 | 0.045607 | -3.84369 |
| PHGDH | 0.520691 | 10.84549 | 2.027366 | 0.045689 | -3.84501 |
| ALPL | 0.639663 | 11.68138 | 2.025742 | 0.045859 | -3.84774 |
| CASP2 | 0.565012 | 11.01534 | 2.024008 | 0.04604 | -3.85065 |
| BNIP3L | -0.51425 | 9.528878 | -2.02132 | 0.046323 | -3.85516 |
| MYB | -0.56171 | 8.010706 | -2.01763 | 0.046714 | -3.86135 |
| C19orf18 | -0.59657 | 6.586451 | -2.01684 | 0.046799 | -3.86268 |
| KLK1 | -0.54745 | 6.445713 | -2.01656 | 0.046828 | -3.86314 |
| ANK2 | -0.55367 | 9.047083 | -2.01472 | 0.047026 | -3.86624 |
| TMEM45B | -0.59595 | 6.102731 | -2.01464 | 0.047034 | -3.86637 |
| MYOZ3 | -0.61493 | 7.140553 | -2.01402 | 0.047101 | -3.86741 |
| BCLAF1 | -0.53323 | 10.65044 | -2.01376 | 0.047128 | -3.86783 |
| LOC154761 | 0.694514 | 6.85778 | 2.012209 | 0.047294 | -3.87043 |
| C13orf16 | 0.577243 | 5.521297 | 2.009336 | 0.047604 | -3.87523 |
| UCA1 | -0.77466 | 6.388063 | -2.00452 | 0.048127 | -3.88326 |
| FAM69A | 0.529762 | 8.979623 | 2.000613 | 0.048555 | -3.88976 |
| CLEC5A | 0.61489 | 8.793037 | 2.000293 | 0.04859 | -3.8903 |
| CSNK1D | 0.58884 | 10.28536 | 2.000044 | 0.048618 | -3.89071 |
| ZBTB48 | 0.550051 | 9.899864 | 1.999818 | 0.048643 | -3.89109 |
| EBNA1BP2 | 0.619992 | 9.144894 | 1.999288 | 0.048701 | -3.89197 |
| FCRL1 | -0.54818 | 6.305675 | -1.99798 | 0.048846 | -3.89414 |
| FGF12 | -0.52337 | 6.967964 | -1.99695 | 0.048959 | -3.89585 |
| FOLR3 | -0.54845 | 5.569371 | -1.99665 | 0.048992 | -3.89634 |
| NYNRIN | -0.61959 | 8.465026 | -1.99019 | 0.049712 | -3.90705 |

**TableS2.** DEP-associated genes from toxicogenomic databases.

|  | DEP Associated genes |
| --- | --- |
| 1 | A4GALT |
| 2 | AADAT |
| 3 | ABCA1 |
| 4 | ABCC2 |
| 5 | ABCC3 |
| 6 | ABCC4 |
| 7 | ABCG1 |
| 8 | ABCG2 |
| 9 | ABCG5 |
| 10 | ABHD14B |
| 11 | ACAA2 |
| 12 | ACACA |
| 13 | ACE |
| 14 | ACHE |
| 15 | ACLY |
| 16 | ACOX1 |
| 17 | ACSBG1 |
| 18 | ACSF2 |
| 19 | ACSS1 |
| 20 | ACTA2 |
| 21 | ADAMTS1 |
| 22 | ADAMTS2 |
| 23 | ADGRE1 |
| 24 | ADGRL1 |
| 25 | ADIPOQ |
| 26 | ADORA1 |
| 27 | ADORA2A |
| 28 | ADORA2B |
| 29 | ADORA3 |
| 30 | ADRA2A |
| 31 | ADRA2B |
| 32 | ADRA2C |
| 33 | ADRB3 |
| 34 | AFP |
| 35 | AHR |
| 36 | AK5 |
| 37 | AKAP1 |
| 38 | AKT1 |
| 39 | AKT2 |
| 40 | AKT3 |
| 41 | ALDH1A1 |
| 42 | ALDH1A2 |
| 43 | ALDH3A1 |
| 44 | ALDOC |
| 45 | ALPL |
| 46 | AMPD3 |
| 47 | ANGPT2 |
| 48 | ANGPTL3 |
| 49 | ANGPTL4 |
| 50 | ANKRD22 |
| 51 | ANKRD50 |
| 52 | ANPEP |
| 53 | ANTXR1 |
| 54 | ANXA8L1 |
| 55 | AP1M2 |
| 56 | APAF1 |
| 57 | APC2 |
| 58 | APLP1 |
| 59 | APLP2 |
| 60 | APOA1 |
| 61 | APOB |
| 62 | APOE |
| 63 | AQP5 |
| 64 | AQP7 |
| 65 | AR |
| 66 | AREG |
| 67 | ARG2 |
| 68 | ARHGAP18 |
| 69 | ARHGAP6 |
| 70 | ARHGEF2 |
| 71 | ARNT2 |
| 72 | ARRDC3 |
| 73 | ARRDC4 |
| 74 | ARXES2 |
| 75 | ASPN |
| 76 | ASTN1 |
| 77 | ASTN2 |
| 78 | ATF3 |
| 79 | ATF4 |
| 80 | ATP10D |
| 81 | ATP1A3 |
| 82 | B3GLCT |
| 83 | BAK1 |
| 84 | BAX |
| 85 | BCAM |
| 86 | BCAT1 |
| 87 | BCHE |
| 88 | BCL2 |
| 89 | BCL2L1 |
| 90 | BCL2L10 |
| 91 | BCL2L15 |
| 92 | BCL6 |
| 93 | BDNF |
| 94 | BEND6 |
| 95 | BEX2 |
| 96 | BHLHE40 |
| 97 | BHLHE41 |
| 98 | BHMT |
| 99 | BID |
| 100 | BNIP3 |
| 101 | BPGM |
| 102 | BRSK1 |
| 103 | BTG2 |
| 104 | BZW1 |
| 105 | C4ORF19 |
| 106 | C5 |
| 107 | CA1 |
| 108 | CA12 |
| 109 | CA2 |
| 110 | CA7 |
| 111 | CA8 |
| 112 | CA9 |
| 113 | CACNG7 |
| 114 | CADM3 |
| 115 | CADPS |
| 116 | CAMK4 |
| 117 | CAMKK1 |
| 118 | CAND2 |
| 119 | CASP2 |
| 120 | CASP3 |
| 121 | CASP8 |
| 122 | CASP9 |
| 123 | CAT |
| 124 | CBLC |
| 125 | CBS |
| 126 | CBX1 |
| 127 | CCDC3 |
| 128 | CCL15-CCL14 |
| 129 | CCL2 |
| 130 | CCL5 |
| 131 | CCN4 |
| 132 | CCND2 |
| 133 | CCNE2 CDK2 CCNE1 |
| 134 | CD14 |
| 135 | CD24 |
| 136 | CD36 |
| 137 | CD59 |
| 138 | CD86 |
| 139 | CD93 |
| 140 | CDH1 |
| 141 | CDHR1 |
| 142 | CDK4 |
| 143 | CDK5R1 |
| 144 | CDKN1A |
| 145 | CDKN1B |
| 146 | CDKN1C |
| 147 | CDKN2A |
| 148 | CDO1 |
| 149 | CEBPA |
| 150 | CELSR3 |
| 151 | CENPM |
| 152 | CERK |
| 153 | CES1 |
| 154 | CES2 |
| 155 | CFAP299 |
| 156 | CGA |
| 157 | CHGA |
| 158 | CHGB |
| 159 | CHRM1 |
| 160 | CHRM2 |
| 161 | CHRM5 |
| 162 | CITED1 |
| 163 | CLDN3 |
| 164 | CLDN8 |
| 165 | CLIP3 |
| 166 | CLSTN3 |
| 167 | CMAH |
| 168 | CMBL |
| 169 | CMTM8 |
| 170 | CNMD |
| 171 | CNR1 |
| 172 | CNTN2 |
| 173 | CNTNAP1 |
| 174 | CNTNAP2 |
| 175 | COL10A1 |
| 176 | COL18A1 |
| 177 | COL1A1 |
| 178 | COL2A1A |
| 179 | COL3A1 |
| 180 | COL6A1 |
| 181 | COL6A2 |
| 182 | COL8A1 |
| 183 | COMT |
| 184 | CPNE8 |
| 185 | CPT1A |
| 186 | CPT1B |
| 187 | CPT2 |
| 188 | CPVL |
| 189 | CPZ |
| 190 | CRABP2 |
| 191 | CREG1 |
| 192 | CRH |
| 193 | CRHR1 |
| 194 | CRMP1 |
| 195 | CRP |
| 196 | CSF1 |
| 197 | CSRNP1 |
| 198 | CTBS |
| 199 | CTH |
| 200 | CTRB1 |
| 201 | CTSB |
| 202 | CTSC |
| 203 | CTSF |
| 204 | CTSG |
| 205 | CTSH |
| 206 | CTSK |
| 207 | CTSL |
| 208 | CTSS |
| 209 | CTSV |
| 210 | CXADR |
| 211 | CXCL1 |
| 212 | CXCL12 |
| 213 | CXCL13 |
| 214 | CXCL5 |
| 215 | CXCL8 |
| 216 | CXCR2 |
| 217 | CXCR4 |
| 218 | CYB5A |
| 219 | CYBA |
| 220 | CYBB |
| 221 | CYCS |
| 222 | CYP11A1 |
| 223 | CYP11B1 |
| 224 | CYP11B2 |
| 225 | CYP17A1 |
| 226 | CYP19A1 |
| 227 | CYP1A1 |
| 228 | CYP1A2 |
| 229 | CYP1B1 |
| 230 | CYP26B1 |
| 231 | CYP2B1 |
| 232 | CYP2C11 |
| 233 | CYP2C19 |
| 234 | CYP2C9 |
| 235 | CYP2D6 |
| 236 | CYP2F2 |
| 237 | CYP2K19 |
| 238 | CYP3A2 |
| 239 | CYP3A4 |
| 240 | CYP3A62 |
| 241 | CYP46A1 |
| 242 | CYP4A1 |
| 243 | D16ERTD472E |
| 244 | DANCR |
| 245 | DCAF12L1 |
| 246 | DCLK3 |
| 247 | DDC |
| 248 | DDIT3 |
| 249 | DDIT4L |
| 250 | DGKB |
| 251 | DGLUCY |
| 252 | DHCR7 |
| 253 | DHRS3 |
| 254 | DKK2 |
| 255 | DNER |
| 256 | DOCK3 |
| 257 | DPF1 |
| 258 | DPYSL3 |
| 259 | DRD1 |
| 260 | DRD3 |
| 261 | DRD4 |
| 262 | DUOXA1 |
| 263 | DUSP4 |
| 264 | DYNC1I1 |
| 265 | EBP |
| 266 | ECE2 |
| 267 | EDN1 |
| 268 | EDN2 |
| 269 | EGFR |
| 270 | EGR1 |
| 271 | ELAVL2 |
| 272 | ELMOD1 |
| 273 | EMB |
| 274 | EMP2 |
| 275 | EMX2 |
| 276 | ENC1 |
| 277 | ENPP3 |
| 278 | EPCAM |
| 279 | EPDR1 |
| 280 | EPHA4 |
| 281 | ERMP1 |
| 282 | ERN1 |
| 283 | ERN2 |
| 284 | ERRFI1 |
| 285 | ESPN |
| 286 | ESR1 |
| 287 | ESR2 |
| 288 | ESRP1 |
| 289 | ESYT1 |
| 290 | ETFDH |
| 291 | ETV5 |
| 292 | EVA1A |
| 293 | EYA2 |
| 294 | F13A1 |
| 295 | F2 |
| 296 | FABP5 |
| 297 | FAH |
| 298 | FAM110C |
| 299 | FAM134B |
| 300 | FAM163A |
| 301 | FAM219A |
| 302 | FAM222A |
| 303 | FAR1 |
| 304 | FASN |
| 305 | FBXL7 |
| 306 | FCGR3 |
| 307 | FEZ1 |
| 308 | FGF11 |
| 309 | FGF2 |
| 310 | FGF5 |
| 311 | FGFR2 |
| 312 | FGG |
| 313 | FHL2 |
| 314 | FKBP1A |
| 315 | FKBP1B |
| 316 | FKBP5 |
| 317 | FLCN |
| 318 | FLG |
| 319 | FOLR1 |
| 320 | FOSB |
| 321 | FOXA1 |
| 322 | FRK |
| 323 | FSD2 |
| 324 | FSHB |
| 325 | FSHR |
| 326 | FXYD3 |
| 327 | FXYD7 |
| 328 | FZD1 |
| 329 | FZD2 |
| 330 | FZD8 |
| 331 | GABRA2 GABRB2 GABRG2 |
| 332 | GABRA2 GABRB3 GABRG2 |
| 333 | GABRB3 GABRA3 GABRG2 |
| 334 | GABRB3 GABRG2 GABRA1 |
| 335 | GABRB3 GABRG2 GABRA5 |
| 336 | GADD45A |
| 337 | GALNT3 |
| 338 | GALNT4 |
| 339 | GALR3 |
| 340 | GANAB |
| 341 | GATA4 |
| 342 | GCLC |
| 343 | GDF15 |
| 344 | GFRA3 |
| 345 | GFRA4 |
| 346 | GGT1 |
| 347 | GGTA1 |
| 348 | GK |
| 349 | GLIPR2 |
| 350 | GLP1R |
| 351 | GLS |
| 352 | GLS2 |
| 353 | GMEB1 |
| 354 | GMNN |
| 355 | GNG2 |
| 356 | GOT1 |
| 357 | GPAM |
| 358 | GPCPD1 |
| 359 | GPD1 |
| 360 | GPER1 |
| 361 | GPI1 |
| 362 | GPR165 |
| 363 | GPR3 |
| 364 | GPR55 |
| 365 | GPRIN2 |
| 366 | GPT |
| 367 | GPX1 |
| 368 | GRAMD1B |
| 369 | GREM2 |
| 370 | GRIN2B |
| 371 | GRM2 |
| 372 | GRM4 |
| 373 | GRM5 |
| 374 | GSK3B |
| 375 | GSR |
| 376 | GSTA3 |
| 377 | H3F3B |
| 378 | HADHA |
| 379 | HADHB |
| 380 | HAS2 |
| 381 | HCK |
| 382 | HEPACAM2 |
| 383 | HK1 |
| 384 | HMGB2 |
| 385 | HMGCR |
| 386 | HMGCS1 |
| 387 | HMGCS2 |
| 388 | HMOX1 |
| 389 | HOXB8 |
| 390 | HOXD3 |
| 391 | HP |
| 392 | HPGDS |
| 393 | HR96 |
| 394 | HSC70 |
| 395 | HSD11B1 |
| 396 | HSD17B11 |
| 397 | HSD17B3 |
| 398 | HSD17B8 |
| 399 | HSD3B1 |
| 400 | HSD3B2 |
| 401 | HSD3B3 |
| 402 | HSDL2 |
| 403 | HSP90B1 |
| 404 | HSPA12A |
| 405 | HSPA5 |
| 406 | HSPB8 |
| 407 | HSPD1 |
| 408 | HTR1A |
| 409 | HTR2A |
| 410 | HTR2B |
| 411 | HTR6 |
| 412 | HTRA1 |
| 413 | HTT |
| 414 | ICA1 |
| 415 | ICAM1 |
| 416 | IDH1 |
| 417 | IDH3A |
| 418 | IDI1-PS1 |
| 419 | IDO1 |
| 420 | IDO2 |
| 421 | IER2 |
| 422 | IFNB1 |
| 423 | IFNG |
| 424 | IGF1 |
| 425 | IGFBP2 |
| 426 | IGFBP5 |
| 427 | IGFBP6 |
| 428 | IGSF5 |
| 429 | IGTP |
| 430 | IL10 |
| 431 | IL13 |
| 432 | IL18 |
| 433 | IL1B |
| 434 | IL4 |
| 435 | IL6 |
| 436 | INHA |
| 437 | INPP5J |
| 438 | INS1 |
| 439 | INSIG1 |
| 440 | INSL3 |
| 441 | IRF2BP2 |
| 442 | IRGM1 |
| 443 | IRGM2 |
| 444 | IRGQ |
| 445 | IRS1 |
| 446 | IRX3 |
| 447 | ISG15 |
| 448 | ITGA11 |
| 449 | ITGA2 |
| 450 | ITGA7 |
| 451 | ITGB3 |
| 452 | ITIH5 |
| 453 | ITPRIP |
| 454 | JAG1 |
| 455 | JAK2 |
| 456 | JAK3 |
| 457 | JUNB |
| 458 | KANK4 |
| 459 | KBTBD11 |
| 460 | KCNK1 |
| 461 | KCTD10 |
| 462 | KCTD11 |
| 463 | KDELR3 |
| 464 | KEAP1 |
| 465 | KIF5A |
| 466 | KIF5C |
| 467 | KLF11 |
| 468 | KLKB1 |
| 469 | KMT5A |
| 470 | KRT18 |
| 471 | KRT19 |
| 472 | L1CAM |
| 473 | LAPTM5 |
| 474 | LATS2 |
| 475 | LBP |
| 476 | LCN2 |
| 477 | LDLR |
| 478 | LEP |
| 479 | LGR4 |
| 480 | LGR6 |
| 481 | LHB |
| 482 | LHCGR |
| 483 | LIPE |
| 484 | LONRF2 |
| 485 | LPCAT1 |
| 486 | LPIN1 |
| 487 | LPL |
| 488 | LRATD1 |
| 489 | LRG1 |
| 490 | LRRC4B |
| 491 | LRRK2 |
| 492 | LRTM2 |
| 493 | LSS |
| 494 | LYNX1 |
| 495 | MAFK |
| 496 | MAGEE1 |
| 497 | MAL2 |
| 498 | MAP2 |
| 499 | MAP6 |
| 500 | MAPK1 |
| 501 | MAPK10 |
| 502 | MAPK14 |
| 503 | MAPK3 |
| 504 | MAPK8 |
| 505 | MAPRE3 |
| 506 | MAPT |
| 507 | MBOAT1 |
| 508 | MBOAT7 |
| 509 | MC2R |
| 510 | MCCC1 |
| 511 | ME3 |
| 512 | METAP1 |
| 513 | MEX3B |
| 514 | MFSD4A |
| 515 | MGLL |
| 516 | MGST1 |
| 517 | MIF |
| 518 | MIR141 |
| 519 | MIR15A |
| 520 | MIR184 |
| 521 | MIR192 |
| 522 | MIR210 |
| 523 | MIR222 |
| 524 | MIR25 |
| 525 | MIR34A |
| 526 | MIR376A1 |
| 527 | MIR376C |
| 528 | MIR572 |
| 529 | MIR590 |
| 530 | MLLT11 |
| 531 | MLXIPL |
| 532 | MME |
| 533 | MMP14 |
| 534 | MMP16 |
| 535 | MMP2 |
| 536 | MMP9 |
| 537 | MPO |
| 538 | MPP2 |
| 539 | MPZL2 |
| 540 | MPZL3 |
| 541 | MRGBP |
| 542 | MRPL1 |
| 543 | MTSS2 |
| 544 | MTUS1 |
| 545 | MUC1 |
| 546 | MUC15 |
| 547 | MVD |
| 548 | MYO10 |
| 549 | MYOCD |
| 550 | NAAA |
| 551 | NAB2 |
| 552 | NACAD |
| 553 | NAIP1 |
| 554 | NAT8L |
| 555 | NCALD |
| 556 | NCEH1 |
| 557 | NCF1 |
| 558 | NCS1 |
| 559 | NDN |
| 560 | NDRG4 |
| 561 | NDUFS1 |
| 562 | NEFH |
| 563 | NEFL |
| 564 | NELL1 |
| 565 | NFE2L2 |
| 566 | NFE2L3 |
| 567 | NGFR |
| 568 | NMRK1 |
| 569 | NNAT |
| 570 | NOL4 |
| 571 | NOS2 |
| 572 | NOTCH4 |
| 573 | NOTUM |
| 574 | NOX4 |
| 575 | NPC1 |
| 576 | NPC1B |
| 577 | NPL |
| 578 | NQO1 |
| 579 | NR1H4 |
| 580 | NR1I2 |
| 581 | NR1I3 |
| 582 | NR3C1 |
| 583 | NR3C2 |
| 584 | NR4A1 |
| 585 | NR4A2 |
| 586 | NR4A3 |
| 587 | NR5A1 |
| 588 | NRBP2 |
| 589 | NRF2 |
| 590 | NRXN2 |
| 591 | NSG1 |
| 592 | NSG2 |
| 593 | NTN4 |
| 594 | NUP210 |
| 595 | NXF3 |
| 596 | OCLN |
| 597 | OLFM1 |
| 598 | OLFML3 |
| 599 | OPRD1 |
| 600 | OPRL1 |
| 601 | OR5M3B |
| 602 | OR6C69B |
| 603 | ORM1 |
| 604 | OSGIN2 |
| 605 | OXT |
| 606 | OXTR |
| 607 | P4HA1 |
| 608 | P4HA2 |
| 609 | PALMD |
| 610 | PAPSS2 |
| 611 | PARP1 |
| 612 | PCCA |
| 613 | PCDHGA11 |
| 614 | PCK1 |
| 615 | PCP4 |
| 616 | PCSK1N |
| 617 | PCSK2 |
| 618 | PCSK5 |
| 619 | PCSK9 |
| 620 | PCYT1A |
| 621 | PDE10A |
| 622 | PDE1B |
| 623 | PDE3A |
| 624 | PDE3B |
| 625 | PDE4B |
| 626 | PDE4D |
| 627 | PDE5A |
| 628 | PDGFB |
| 629 | PDGFC |
| 630 | PDK1 |
| 631 | PDK3 |
| 632 | PDK4 |
| 633 | PDZK1IP1 |
| 634 | PDZRN3 |
| 635 | PEG13 |
| 636 | PER1 |
| 637 | PEX5L |
| 638 | PFKP |
| 639 | PFN2 |
| 640 | PGAM1 |
| 641 | PGR |
| 642 | PHOSPHO1 |
| 643 | PHTF1 |
| 644 | PIK3CD PIK3R1 |
| 645 | PIK3R1 |
| 646 | PIN1 |
| 647 | PLA2G2A |
| 648 | PLAT |
| 649 | PLAU |
| 650 | PLCG1 |
| 651 | PLCL2 |
| 652 | PLEKHA6 |
| 653 | PLEKHB1 |
| 654 | PLET1 |
| 655 | PLIN1 |
| 656 | PLIN2 |
| 657 | PLIN5 |
| 658 | PLPP2 |
| 659 | PLPP3 |
| 660 | PLPP7 |
| 661 | PLPPR3 |
| 662 | PLS1 |
| 663 | PLTP |
| 664 | PLXNC1 |
| 665 | POF1B |
| 666 | PPARA |
| 667 | PPARB |
| 668 | PPARG |
| 669 | PPARGC1A |
| 670 | PPP2R2B |
| 671 | PRDM1 |
| 672 | PREP |
| 673 | PRIMA1 |
| 674 | PRKAR2B |
| 675 | PRLR |
| 676 | PROCR |
| 677 | PROM1 |
| 678 | PRPH |
| 679 | PRSS23 |
| 680 | PRSS8 |
| 681 | PRXL2A |
| 682 | PSMB1 |
| 683 | PTGDS |
| 684 | PTGER2 |
| 685 | PTGES |
| 686 | PTGR1 |
| 687 | PTGS2 |
| 688 | PTK2 |
| 689 | PTPN5 |
| 690 | PTPRN |
| 691 | PTX3 |
| 692 | QSOX1 |
| 693 | RAB25 |
| 694 | RAB3B |
| 695 | RAB6B |
| 696 | RACK1 |
| 697 | RALGDS |
| 698 | RAMP1 |
| 699 | RARG |
| 700 | RARRES2 |
| 701 | RASGEF1B |
| 702 | RASL11B |
| 703 | RASSF4 |
| 704 | RBP4 |
| 705 | RBPMS |
| 706 | RCAN2 |
| 707 | RELA |
| 708 | RESP18 |
| 709 | RETSAT |
| 710 | RGS2 |
| 711 | RGS4 |
| 712 | RHOX12 |
| 713 | RNF125 |
| 714 | RNF128 |
| 715 | RNF145 |
| 716 | RNF180 |
| 717 | RNF183 |
| 718 | RPS16 |
| 719 | RPS6KA2 |
| 720 | RRAS2 |
| 721 | RTN1 |
| 722 | RTN4R |
| 723 | RXRA |
| 724 | RXRB |
| 725 | RXRG |
| 726 | SAA3 |
| 727 | SAMD4B |
| 728 | SCARA5 |
| 729 | SCARB1 |
| 730 | SCD |
| 731 | SCD1 |
| 732 | SCD4 |
| 733 | SCG3 |
| 734 | SCG5 |
| 735 | SCN3B |
| 736 | SCNN1B |
| 737 | SCRN1 |
| 738 | SDR16C5 |
| 739 | SECTM1 |
| 740 | SELENOP |
| 741 | SEMA7A |
| 742 | SERPINB1A |
| 743 | SERPINB2 |
| 744 | SERPINB5 |
| 745 | SERPINB6B |
| 746 | SERPINB9E |
| 747 | SERPINE1 |
| 748 | SERPINF1 |
| 749 | SFRP1 |
| 750 | SFRP2 |
| 751 | SH3BP4 |
| 752 | SH3BP5 |
| 753 | SH3BP5L |
| 754 | SH3GL3 |
| 755 | SH3TC2 |
| 756 | SH3YL1 |
| 757 | SHBG |
| 758 | SHC2 |
| 759 | SIK1 |
| 760 | SIX4 |
| 761 | SLAIN1 |
| 762 | SLC12A2 |
| 763 | SLC12A7 |
| 764 | SLC13A2 |
| 765 | SLC13A5 |
| 766 | SLC16A3 |
| 767 | SLC16A6 |
| 768 | SLC1A5 |
| 769 | SLC1A6 |
| 770 | SLC20A1 |
| 771 | SLC24A3 |
| 772 | SLC25A15 |
| 773 | SLC25A51 |
| 774 | SLC27A1 |
| 775 | SLC27A4 |
| 776 | SLC27A6 |
| 777 | SLC29A4 |
| 778 | SLC2A1 |
| 779 | SLC2A2 |
| 780 | SLC2A3 |
| 781 | SLC2A4 |
| 782 | SLC34A2 |
| 783 | SLC36A2 |
| 784 | SLC38A3 |
| 785 | SLC38A5 |
| 786 | SLC44A3 |
| 787 | SLC44A4 |
| 788 | SLC5A3 |
| 789 | SLC5A7 |
| 790 | SLC6A2 |
| 791 | SLC6A7 |
| 792 | SLC7A7 |
| 793 | SLC7A8 |
| 794 | SLC8A1 |
| 795 | SLC9A3R1 |
| 796 | SLCO1D1 |
| 797 | SLCO2A1 |
| 798 | SLFN2 |
| 799 | SLIT3 |
| 800 | SMAD3 |
| 801 | SMAD3A |
| 802 | SMAD7 |
| 803 | SMIM24 |
| 804 | SMPD3 |
| 805 | SNHG11 |
| 806 | SNN |
| 807 | SNTB1 |
| 808 | SOAT1 |
| 809 | SOCS3 |
| 810 | SOD1 |
| 811 | SORD |
| 812 | SOWAHB |
| 813 | SOX9 |
| 814 | SOX9A |
| 815 | SPATA13 |
| 816 | SPECC1 |
| 817 | SPINT1 |
| 818 | SPINT2 |
| 819 | SQLE |
| 820 | SREBF1 |
| 821 | SREBF2 |
| 822 | SRPX2 |
| 823 | ST6GALNAC2 |
| 824 | STAR |
| 825 | STK32C |
| 826 | STMN4 |
| 827 | STON2 |
| 828 | SUCLG2 |
| 829 | SULF1 |
| 830 | SYT11 |
| 831 | SYT5 |
| 832 | TACR2 |
| 833 | TACSTD2 |
| 834 | TARDBP |
| 835 | TBL1XR1 |
| 836 | TBX2 |
| 837 | TBXAS1 |
| 838 | TCF23 |
| 839 | TCHHL1 |
| 840 | TCP11X2 |
| 841 | TCTE1 |
| 842 | TDRP |
| 843 | TFAP2B |
| 844 | TFRC |
| 845 | TGFA |
| 846 | TGFB1 |
| 847 | TGFB3 |
| 848 | TGFBR1A |
| 849 | TGM2 |
| 850 | THBS3 |
| 851 | THBS4 |
| 852 | THSD4 |
| 853 | THSD7B |
| 854 | TIMP1 |
| 855 | TJP1 |
| 856 | TLNRD1 |
| 857 | TLR2 |
| 858 | TLR4 |
| 859 | TLR9 |
| 860 | TMC4 |
| 861 | TMEFF2 |
| 862 | TMEM130 |
| 863 | TMEM184A |
| 864 | TMEM251 |
| 865 | TMEM30B |
| 866 | TMEM35A |
| 867 | TMEM47 |
| 868 | TMEM50B |
| 869 | TMEM63C |
| 870 | TMSB15B2 |
| 871 | TMSB15L |
| 872 | TMX1 |
| 873 | TNF |
| 874 | TNFAIP6 |
| 875 | TNFRSF11B |
| 876 | TNFRSF21 |
| 877 | TNKS2 |
| 878 | TOB2 |
| 879 | TOM1L1 |
| 880 | TOR4A |
| 881 | TP53 |
| 882 | TPH1 |
| 883 | TPI1 |
| 884 | TRAP1 |
| 885 | TRARG1 |
| 886 | TRIB1 |
| 887 | TRIM6 |
| 888 | TRPA1 |
| 889 | TRPC6 |
| 890 | TSHR |
| 891 | TSPAN1 |
| 892 | TSPO |
| 893 | TTYH1 |
| 894 | TTYH3 |
| 895 | TUBB2B |
| 896 | TUBB3 |
| 897 | TUBB4A |
| 898 | TXNIP |
| 899 | TXNRD1 |
| 900 | UBASH3B |
| 901 | UBXN11 |
| 902 | UCP1 |
| 903 | UNC5D |
| 904 | USP10 |
| 905 | USP13 |
| 906 | USP18 |
| 907 | VAMP5 |
| 908 | VEGFA |
| 909 | VEGFD |
| 910 | VGLL4 |
| 911 | VOPP1 |
| 912 | VTG2 |
| 913 | VTRNA2-1 |
| 914 | WDR92 |
| 915 | WFDC2 |
| 916 | WFS1 |
| 917 | WNT4 |
| 918 | WNT6 |
| 919 | WNT9B |
| 920 | WT1 |
| 921 | XBP1 |
| 922 | XDH |
| 923 | ZBTB8B |
| 924 | ZCCHC12 |
| 925 | ZDHHC23 |
| 926 | ZFAT |
| 927 | ZFP280B |
| 928 | ZFP330 |
| 929 | ZFP467 |
| 930 | ZFP503 |
| 931 | ZFP791 |
| 932 | ZP2 |

**TableS3.** Overlapping genes between DEP-associated genes and OS-specific DEGs.

|  | 45 overlapping genes |
| --- | --- |
| 1 | ABCA1 |
| 2 | ADIPOQ |
| 3 | ALPL |
| 4 | APOB |
| 5 | AQP7 |
| 6 | BCL2L15 |
| 7 | CA1 |
| 8 | CASP2 |
| 9 | CDKN2A |
| 10 | COL10A1 |
| 11 | COL18A1 |
| 12 | COL3A1 |
| 13 | COL6A2 |
| 14 | CRABP2 |
| 15 | CTSB |
| 16 | FAH |
| 17 | GALNT4 |
| 18 | GLS2 |
| 19 | HAS2 |
| 20 | HMGCS1 |
| 21 | HSP90B1 |
| 22 | HSPA5 |
| 23 | ISG15 |
| 24 | ITGA7 |
| 25 | ITBG3 |
| 26 | KEP1 |
| 27 | LCN2 |
| 28 | LDLR |
| 29 | MLLT11 |
| 30 | MPO |
| 31 | P4HA2 |
| 32 | PER1 |
| 33 | PLAT |
| 34 | PPARG |
| 35 | PPKAR2B |
| 36 | PRSS23 |
| 37 | RTN1 |
| 38 | SCL29A4 |
| 39 | SLC2A4 |
| 40 | SCL7A8 |
| 41 | TGFB3 |
| 42 | THBS3 |
| 43 | THBS4 |
| 44 | THSD7B |
| 45 | TUBB3 |
